# Genome-wide association studies for corneal and refractive astigmatism in UK Biobank demonstrate a shared role for myopia susceptibility loci

**DOI:** 10.1007/s00439-018-1942-8

**Published:** 2018-10-10

**Authors:** Rupal L. Shah, Jeremy A. Guggenheim, UK Biobank Eye and Vision Consortium

**Affiliations:** School of Optometry and Vision Sciences, Cardiff University, Cardiff, CF24 4HQ UK

## Abstract

Previous studies have suggested that naturally occurring genetic variation contributes to the risk of astigmatism. The purpose of this investigation was to identify genetic markers associated with corneal and refractive astigmatism in a large-scale European ancestry cohort (UK Biobank) who underwent keratometry and autorefraction at an assessment centre. Genome-wide association studies for corneal and refractive astigmatism were performed in individuals of European ancestry (*N* = 86,335 and 88,005 respectively), with the mean corneal astigmatism or refractive astigmatism in fellow eyes analysed as a quantitative trait (dependent variable). Genetic correlation between the two traits was calculated using LD Score regression. Gene-based and gene-set tests were carried out using MAGMA. Single marker-based association tests for corneal astigmatism identified four genome-wide significant loci (*P* < 5 × 10^−8^) near the genes *ZC3H11B* (1q41), *LINC00340* (6p22.3), *HERC2*/*OCA2* (15q13.1) and *NPLOC4*/*TSPAN10* (17q25.3). Three of these loci also demonstrated genome-wide significant association with refractive astigmatism: *LINC00340, HERC2*/*OCA2* and *NPLOC4*/*TSPAN10*. The genetic correlation between corneal and refractive astigmatism was 0.85 (standard error = 0.068, *P* = 1.37 × 10^−35^). Here, we have undertaken the largest genome-wide association studies for corneal and refractive astigmatism to date and identified four novel loci for corneal astigmatism, two of which were also novel loci for refractive astigmatism. These loci have previously demonstrated association with axial length (*ZC3H11B*), myopia (*NPLOC4*), spherical equivalent refractive error (*LINC00340*) and eye colour (*HERC2*). The shared role of these novel candidate genes for astigmatism lends further support to the shared genetic susceptibility of myopia and astigmatism.

**Electronic supplementary material:**

The online version of this article (10.1007/s00439-018-1942-8) contains supplementary material, which is available to authorized users.

## Introduction

Astigmatism occurs when the eye fails to bring light from a point source object to a single point focus on the retina, resulting in impaired vision. If uncorrected in childhood, astigmatism is a risk factor for amblyopia development (Read et al. 2007; Harvey 2009). In the human eye, the two major sources of astigmatism are the cornea and the crystalline lens. Astigmatism can be described as either “refractive”, which encompasses all contributing sources, or “corneal”, which is restricted to the corneal component, the major contributing source in most cases of moderate and high astigmatism (Read et al. 2007).

Family- and twin-based studies have previously reported that genetic factors make a notable contribution to the development of corneal and refractive astigmatism, with the proportion of trait variance attributable to genetic effects (heritability) estimated at 50–65% (Hammond et al. 2001; Kim et al. 2013; Dirani et al. 2006; Clementi et al. 1998; Grjibovski et al. 2006). Genome-wide association studies (GWAS) for refractive astigmatism and corneal astigmatism have identified a single locus for each; in the promoter of *PDGFRA* (4q12) for corneal astigmatism (Fan et al. 2011; Guggenheim et al. 2013; Shah et al. 2018) and near *NRXN1* (2p16.3) for refractive astigmatism (Li et al. 2015). Additional loci demonstrating suggestive association (*P* < 1 × 10^−5^) for refractive astigmatism have been identified near the genes *VAX2, TOX* and *LINC00340* (Li et al. 2015; Lopes et al. 2013). In contrast, more than a hundred markers have shown genome-wide significant association (*P* < 5 × 10^−8^) for the highly heritable refractive traits spherical equivalent and myopia in GWAS studies (Kiefer et al. 2013; Pickrell et al. 2016; Verhoeven et al. 2013; Tedja et al. 2018).

This paucity of genome-wide significant markers identified in GWAS for astigmatism may be due to any of the following reasons. Firstly, limitations of the approach used to define astigmatism in previous studies, such as the use of an arbitrary threshold for assigning case/control status. Secondly, the effects of age may not have been taken into account fully, since changes in both the prevalence and direction (axis) of astigmatism occur across the lifespan (Sanfilippo et al. 2015; Schuster et al. 2017). Thirdly, there may be a major role for rare variants, i.e. risk alleles with a minor allele frequency (MAF) < 1%, which are typically excluded from GWAS analyses, and fourthly, the causal markers may have extremely small effect sizes, thus rendering previous studies underpowered to detect associated markers because of their insufficient sample size. The largest study published to date was a GWAS meta-analysis for refractive astigmatism performed by Li et al. (2015), which had a sample of 45,931 individuals, including 36,636 individuals of European ancestry and 9295 individuals of Asian ancestry.

The availability of genotype data for the UK Biobank cohort, approximately 23% of whom had data on corneal and/or refractive astigmatism from non-cycloplegic autorefraction, provided an opportunity to identify genetic markers associated with corneal or refractive astigmatism using a comprehensive approach and at a larger scale than had been possible previously.

## Methods

### UK biobank sample

UK Biobank is a large prospective study following the health and wellbeing of approximately 500,000 participants resident in the UK aged between 40 and 69 years-old at the baseline recruitment visit (during the period 2006–2010). UK Biobank received ethical approval from the NHS Research Ethics Committee (Reference: 11/NW/0382). Baseline assessment was undertaken at 1 of 22 assessment centres distributed across the UK (Allen et al. 2014; Sudlow et al. 2015). Approximately 20,000 participants also attended the first repeat assessment visit (during the period 2012 to 2013). Demographic information and medical history were ascertained through touch-screen questionnaires. Participants also underwent a wide range of physical and cognitive assessments, including blood sampling (for DNA) and, for participants recruited towards the end of the recruitment period, an ophthalmic examination. Phenotyping, genotyping and imputation were carried out by members of the UK Biobank team.

### Phenotypes

119,806 participants had keratometry readings taken for at least 1 eye using the Tomey RC 5000 autorefractor-keratometer (Tomey Corp., Nagoya, Japan). Up to 6 measurements were taken for each eye using 6-mm diameter keratometry mires, from which corneal astigmatism was derived (see below). 130,521 participants had non-cycloplegic autorefraction performed for at least one eye using the same auto autorefractor-keratometer, with up to ten measurements taken for each eye. In all instances, participants were required to remove contact lenses, if worn. Refractive astigmatism was derived from the autorefraction cylindrical power. Spherical equivalent was recorded as the spherical power plus half of the cylindrical power from autorefraction. All keratometry/autorefractor measurements flagged with an error code “E” (indicating “Lower reliability data”) were recoded as missing before taking the mean trait values for each eye individually across assessment centre visits, then the mean of both eyes for each individual. The mean corneal astigmatism and mean refractive astigmatism for each individual were also categorised as dichotomous variables using a grid of thresholds to define case/control status, from 0.50 to 1.50 D, in 0.25 D steps. After the exclusion of unreliable readings, 119,799 participants had measures for corneal astigmatism, and 130,459 participants had measures for refractive astigmatism and spherical equivalent refractive error.

### Genotyping and imputation

Participant DNA samples were genotyped by UK Biobank researchers at approximately 800,000 genetic markers using one of two genotyping arrays, the UK BiLEVE Axiom array or the UK Biobank Axiom array. Genetic data were released in two waves. In the UK Biobank “Interim 150K” release, data were made available for 152,725 samples imputed at 72,355,689 markers using IMPUTE2 (Howie et al. 2011) with a merged 1000 Genomes Project Phase 3 and UK10K Project haplotype reference panel (Davies et al. 2016; Wain et al. 2015). Further details of the imputation protocol can be found at http://biobank.ctsu.ox.ac.uk/crystal/refer.cgi?id=157020. As detailed below, of these 152,725 genotyped participants, 141,751 were of European ancestry based on principal components analysis (PCA), and were non-outliers for heterozygosity. Data from this UK Biobank “Interim 150K” release were used for SNP-heritability estimation.

The PCA for data from participants in the “Interim 150K” release was carried out as follows. Following Abraham and Inouye (2014), genotype data for 1397 individuals from release #3 of phase 3 of the HapMap project were downloaded, and related individuals were excluded based on the pedigree file “relationships_w_pops_041510.txt”. For each HapMap3 ancestry group separately, SNPs with minor allele frequency (MAF) < 1%, missingness > 1%, Hardy–Weinberg equilibrium (HWE) *P* < 5 × 10E−6 and non-autosomal variants were removed, as were individuals with missingness > 1%, and genomic regions of high linkage disequilibrium (LD) and/or known inversions (chr5: 44–51.5 Mb, chr6: 25–33.5 Mb, chr8: 8–12 Mb, chr11: 45–57 Mb). The remaining SNPs were intersected with those included on the two genotyping arrays used by UK Biobank (as listed in the files, “Axiom_UKB_WCSG.na34.annot2.csv” and “Axiom_UKBiLEVE.na34.annot.csv” from the Affymetrix website) and LD-pruned (Chang et al. 2015) using the Plink 1.9 command—indep-pairwise 1000 10 0.1, resulting in a set of 56,401 common, successfully genotyped variants in HWE and low LD present in both UK Biobank and HapMap3. With the genotype data for these SNPs, principal components analysis was carried out for 1114 unrelated HapMap3 individuals who clustered according to their assigned ancestry, using the smartpca program from the Eigensoft package (Price et al. 2006). PCAs were projected onto UK Biobank participants and individuals of European ancestry were defined as lying within four standard deviations of the mean for the first 20 PCs. Of the 152,729 individuals included in the UK Biobank genetic data interim release, 142,126 (93.1%) were identified as having European ancestry. Excluding heterozygosity outliers (defined as lying outside the mean ±4 standard deviation heterozygosity range) from this European ancestry sample left 141,751 individuals.

The second wave (July 2017 release) of genetic data released from the UK Biobank consisted of imputed genotype information for all 488,377 participants whose data passed quality control filters (Bycroft et al. 2017). This release of genetic data included all available individuals from the “Interim 150K” data release. Briefly, imputation was carried out by Bycroft et al. (2017) using IMPUTE4, an updated version of IMPUTE2 (Howie et al. 2011; Bycroft et al. 2017) with a reference panel comprising of the Haplotype Reference Consortium (HRC) and a merged 1000 Genomes Project Phase 3 and UK10K Project haplotype reference panel (Bycroft et al. 2017; McCarthy et al. 2016; The UK 10K Consortium et al. 2015). Due to uncertainty about the reliability of the 1000 Genomes and UK10K imputations, for the present work only the ~ 40 million markers present in the HRC imputation panel were utilised. All markers were mapped to NCBI human genome build 37 (hg19/GRCh37) coordinates.

Of the 488,377 genotyped participants, 409,728 were classified as a “White British-ancestry subset” by Bycroft et al. (2017). These 409,728 individuals self-reported White British ethnicity and clustered together with other individuals of White British-ancestry in a PCA analysis (Bycroft et al. 2017). After excluding heterozygosity outliers (heterozygosity within four standard deviations of the mean of the White British-ancestry subset) data for these individuals from the second wave of genetic data (July 2017 release) were used for the GWAS analyses.

### Exclusion criteria

To minimise the effects of ocular pathology or surgery affecting keratometry/autorefraction readings, individuals were excluded from analyses if at any visit, they self-reported having had any injury or trauma resulting in loss of vision, cataract extraction/lens implant, surgery for glaucoma or high eye-pressure or trabeculectomy, refractive laser eye surgery, corneal graft surgery, or any eye surgery in the last four weeks. Individuals responding “Don’t know” to the latter four questions were also excluded. For analyses of refractive astigmatism, individuals were further excluded if they self-reported having had: cataract or “other serious eye condition”, any eye surgery or retinal operation/vitrectomy. The UK Biobank study did not include specific questions regarding contact lens wear, thus individuals could not be excluded from analyses for reasons pertaining to the wear of specific contact lens types such as orthokeratology lenses. However, orthokeratology lenswear in the 40–70 year age group is rare in the UK (Morgan 2007). Individuals whose self-reported and genetically inferred sex differed were also omitted from the analyses.

### “High-confidence” markers

For the mixed model analyses carried out using BOLT and GCTA (see below) a set of approximately 890,000 “high-confidence” markers in weak LD was generated using PLINK 2.0 (Purcell et al. 2007; Chang et al. 2015). All markers with an “rs” prefix that were directly genotyped or imputed in at least 99% of individuals, with MAF > 0.005 and imputation quality (INFO) > 0.90 were LD-pruned (using the command–indep-pairwise 50 5 0.1) to obtain list of markers for creating genetic relationship matrices (GRMs). Of these “high-confidence” markers, approximately 23% had been directly genotyped.

### SNP-heritability estimation

Using the “high-confidence” markers, the PLINK 1.9 command—make-grm-bin—was used to create separate GRMs for the analysis of corneal astigmatism and the refractive phenotypes (refractive astigmatism and spherical equivalent). All individuals in the “Interim 150K” release dataset who, after exclusions, had information available for the respective phenotype were included in the initial GRMs, which were subsequently restricted to unrelated individuals based on a pairwise relatedness threshold of 0.025 (approximately equivalent to third degree relatives) (Yang et al. 2011b). If pairs of individuals had a relatedness coefficient greater than this cut-off, one individual from this pair was removed. The final GRMs for corneal astigmatism and for the two refractive phenotypes had sample sizes of 27,737 and 28,403 unrelated individuals, respectively. SNP-heritability (*h*^2^_SNP_) estimates were obtained using the default additive effects model in GCTA (Yang et al. 2011a). Corneal astigmatism, refractive astigmatism and spherical equivalent were considered as continuous traits, or as dichotomous traits defined using each of the different case thresholds examined (0.50 D–1.50 D, in 0.25 D steps for corneal and refractive astigmatism; and − 0.50 D to − 1.50 D, in 0.25 D steps for spherical equivalent). For dichotomous traits, transformation to the liability scale was performed by GCTA, as described by Lee et al. (2011). Approximate population prevalence estimates were obtained from the full sample of UK Biobank individuals with valid phenotype measures, irrespective of ancestry or exclusion criteria.

Additional SNP-heritability estimates were obtained in an attempt to partition SNP-heritability into separate additive and dominance components. GCTA-GREMLd (Zhu et al. 2015) was used to generate additive and dominance GRMs for corneal astigmatism and the refractive phenotypes using the same unrelated individuals as used previously (*N* = 27,737 and 28,403 respectively). Both additive and dominance GRMs for each trait were included in a joint GCTA analysis to ascertain the partitioned contributions of additive and dominance effects to the respective phenotypic variances. All traits were coded as continuous variables for these GREMLd analyses.

### Genome-wide association studies (GWAS)

Genome-wide single marker association tests were undertaken for corneal astigmatism (*N* = 86,335) and for refractive astigmatism (*N* = 88,005) using individuals with genetic data made available in the UK Biobank second data release (July 2017 release). Corneal and refractive astigmatism were considered as continuous traits using the standard “infinitesimal” mixed linear model approach implemented in BOLT-LMM v2.3. In BOLT-LMM, GRMs constructed using the “high-confidence” markers were used to account for residual population structure and cryptic relatedness (Yang et al. 2014; Loh et al. 2015, 2018); therefore, related individuals were not excluded from the BOLT-LMM analyses. Regional association plots for genome-wide significant loci were created using LocusZoom (Pruim et al. 2010). Sensitivity analyses were performed using PLINK 2.0, with corneal and refractive astigmatism considered as continuous traits (as in BOLT-LMM analyses) and then by classifying astigmatism as a dichotomous trait using a threshold value of astigmatism ≥ 1.00 D to define case status. As PLINK 2.0 used linear/logistic regression methods to run association analyses, these tests were restricted to unrelated individuals. A pairwise relatedness threshold of 0.025 was applied to remove one of each pair of related individuals from these PLINK 2.0 analyses. For all GWAS analyses, participants’ spherical equivalent refractive error and age at the assessment visit were included as quantitative covariates (using average values for participants who attended more than one visit). Genotyping array (UK BiLEVE or UK Biobank) and sex (female or male) were included as discrete covariates. Following Fan et al. (2016), markers with missingness > 0.01, MAF < 0.01, HWE test *P* value < 1 × 10^−6^ or INFO < 0.4 were excluded, along with samples with missingness > 0.05.

For loci achieving the genome-wide significance threshold of *P* < 5 × 10^−8^, previously identified associations with other ocular traits were identified via the NHGRI-EBI catalogue of published genome-wide association studies (MacArthur et al. 2017).

### Conditional analysis

To ascertain whether loci achieving genome-wide significant association were driven by a single causal marker or by multiple causal markers in the region, conditional analysis was performed using GCTA-COJO (Yang et al. 2012). The marker demonstrating the strongest degree of association at a genome-wide significant locus was included as a covariate in the association test model and the association analysis repeated for all markers within ± 1000 kb of this marker. The association signals obtained in the conditional analysis will be greatly diminished compared to the original analysis in the event of there being only a single causal locus. In the presence of multiple causal markers at a locus, markers tagging additional causal markers, independent of the conditioned marker will continue to demonstrate significant association in the GCTA-COJO analysis.

### Genomic inflation of GWAS summary statistics

The genomic inflation factor (*λ*_GC_) was determined by dividing the median observed *χ*^2^ test statistic by 0.456 (Devlin et al. 2001). However, since *λ*_GC_ determined using this method can be overly conservative in cases of true polygenicity (Bulik-Sullivan et al. 2015b), we also calculated the intercept from an LD Score (LDSC) regression analysis, using European ancestry individuals from the 1000 Genomes Project for the reference LD scores, as described by Bulik-Sullivan et al. (2015b).

### Genetic effect sizes in male vs. female participants

To test for a gender-related difference in effect size for the lead variants associated with corneal astigmatism and refractive astigmatism in the GWAS analyses, linear regression analyses were carried out separately in males and females for astigmatism coded as a continuous trait. The genetic effect sizes (beta coefficients for the SNP effect from the regression analyses) were then compared between males and females, as described by Winkler et al. (2014). The sex-specific linear regression analyses were carried out using the same set of White British unrelated individuals as for the PLINK 2.0 analyses of astigmatism described above. Age, spherical equivalent refractive error, and a binary indicator for genotyping array were included as covariates. To account for multiple testing (7 variants tested in total), a *P* value < 0.05/7 = 0.007 was taken as the threshold for declaring a difference in effect size between males and females.

### Gene-based and gene-set tests

Gene-based and gene-set tests were performed using the summary statistics results from GWAS in MAGMA v1.06 (de Leeuw et al. 2015). Genes were defined according to NCBI build 37 (hg19/GRCh37) coordinates, with the inclusion of a 50 kb flanking region added to the transcription start/stop positions. These flanking regions were added to genomic regions as polymorphisms in these 5′ and 3′ regions often influence gene regulation and expression not only for the nearest gene but for other nearby genes too (Corradin et al. 2016; Guo and Jamison 2005; Brodie et al. 2016; Schork et al. 2013). MAGMA estimates LD patterns for each gene using an ancestry-matched reference file; specifically the reference files composed of data for the 503 unrelated individuals of European ancestry from Phase 3 of the 1000 Genomes Project. For the gene-based tests, multiple testing was accounted for by applying a false discovery rate threshold of 5%.

Gene-set tests in MAGMA were performed using a “competitive” approach whereby the test statistics for all genes within a particular gene-set (e.g. a biological pathway) were combined to obtain a joint association statistic. This statistic was compared against that for all other genes not in that set whilst accounting for the number of SNPs within each gene, gene density and differential sample size (unequal sample size contributing to each gene) (de Leeuw et al. 2015, 2016). Gene-sets were defined using the Molecular Signatures Database (MSigDB) (Subramanian et al. 2005). Gene definitions and their respective association signals for genes contributing to gene-sets were taken from the MAGMA gene-based analyses with the aim of identifying potential biological processes that may be influenced by these markers. Multiple testing was accounted for by applying a false discovery rate threshold of 5%.

### SNP-heritability and genetic correlation analyses using GWAS summary statistics

Using LDSC (Bulik-Sullivan et al. 2015a, b), SNP-heritabilities of corneal astigmatism and refractive astigmatism were quantified using summary statistics from the single marker association tests conducted using BOLT-LMM. Genetic correlations between pairs of the three refractive error traits: corneal astigmatism, refractive astigmatism and spherical equivalent were also quantified using this method. Summary statistics for spherical equivalent were obtained from single marker association tests conducted using BOLT-LMM for the same variants and individuals as performed for refractive astigmatism. In all instances, the reference LD scores used were the same as those utilised when calculating the intercepts during LD Score regression analyses.

### Phenotypic correlation

Pearson correlation coefficients were calculated for all unrelated individuals included in the single marker GWAS analyses who had data available for all refractive error traits investigated (i.e. corneal astigmatism, refractive astigmatism and spherical equivalent; *N* = 63,466).

## Results

### Participant demographics

Analyses were carried out in a sample of UK Biobank participants with a mean age of 58.2 years (standard deviation: 7.9 years; 25th and 75th percentiles: 52.25 and 64.50 years) of White British/European ancestry, based on genetic principal components analysis. Approximately 54% of the sample were female, and the mean spherical equivalent refractive error was − 0.29 D (standard deviation: 2.72 D; 25th and 75th percentiles: − 1.23 and + 1.13 D). Approximately 4.0% of the participants had high myopia (defined as a refractive error averaged between the two eyes ≤ − 6.00 D) and 18.0% of the sample had anisometropia of at least 1.00 D. The level of corneal astigmatism was relatively constant across the age spectrum of the participants, while the level of refractive astigmatism increased with age (Online Resource 1). Full details of the refractive error and demographic characteristics of the participants have been reported by Cumberland et al. (2015).

### Determining optimal trait definitions based on SNP-heritability

Astigmatism has often been analysed as a dichotomous trait in genetic studies; however, the choice of the threshold used to define case/control status has varied from study to study (Fan et al. 2011; Vitale et al. 2008; Li et al. 2015; Hammond et al. 2001; Dirani et al. 2010; Quek et al. 2004; He et al. 2004; Huynh et al. 2007; Shah et al. 2018). In an attempt to determine an optimal trait definition for detecting commonly occurring genetic markers with additive effects on astigmatism, we calculated SNP-heritability estimates with GCTA for corneal astigmatism and refractive astigmatism (and, for comparison, spherical equivalent) classified either as continuous or dichotomous traits, and using a grid of case thresholds for the latter (namely, 0.50, 0.75, 1.00, 1.25 and 1.50 D of astigmatism; or − 0.50, − 0.75, − 1.00, − 1.25 and − 1.50 D of spherical equivalent). Following previous precedents (Schulze and McMahon 2004; Corvin et al. 2010; Koran et al. 2014), this approach was predicated on the assumption that the trait definition capturing the greatest SNP-heritability would be the one most likely to highlight genome-wide significant loci in subsequent GWAS of these traits.

**Fig. 1:**
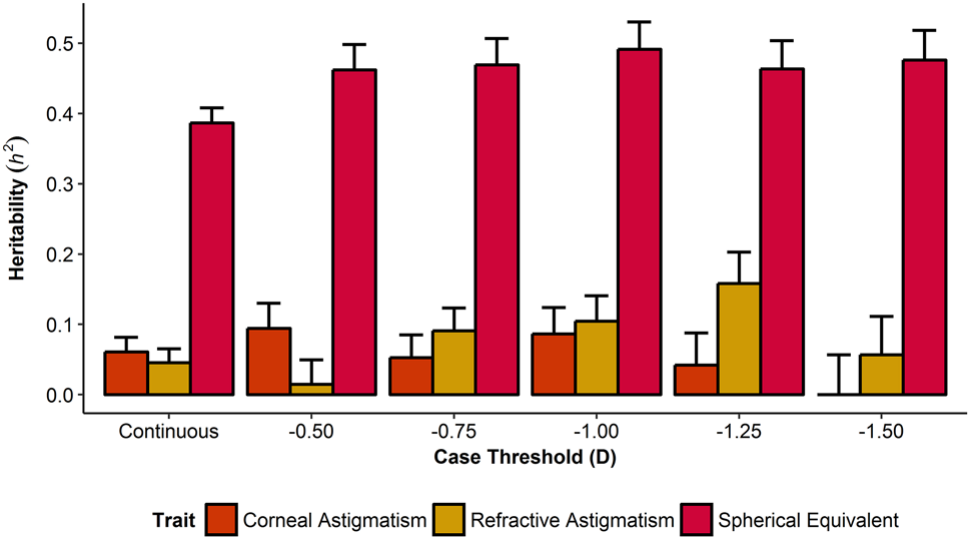
Estimates of SNP-heritability (*h*^2^_SNP_) using GCTA. Error bars represent the standard error of the *h*^2^_SNP_ estimate

For corneal astigmatism, SNP-heritability was greatest using a case-definition threshold of 0.50 D (*h*^2^_SNP_ = 0.094) and negligible for a case threshold of 1.50 D (Fig. 1, Online Resource 2). However, there appeared to be no statistically meaningful difference in SNP-heritability across the range of trait definitions tested since all of the standard errors overlapped (Fig. 1). For refractive astigmatism, SNP-heritability estimates were generally higher than those for corneal astigmatism (*h*^2^_SNP_: 0.015–0.158; Fig. 1, Online Resource 2). Using case-definition thresholds of increasing magnitude between 0.50 D and 1.25 D inclusive yielded increasing SNP-heritability estimates, although the overlapping standard errors meant that, again, there was no statistical support for meaningful differences across the range of case thresholds tested. SNP-heritability estimates for spherical equivalent refractive error were much larger than those for the astigmatism traits (*h*^2^_SNP_: 0.462–0.491; Fig. 1, Online Resource 2).

When astigmatism and spherical equivalent were modelled as continuous traits, the estimates of SNP-heritability were numerically lower, but with much narrower standard errors, compared to when they were modelled as dichotomous traits: continuous trait *h*^2^_SNP_ (SE): corneal astigmatism = 0.061 (0.021); refractive astigmatism = 0.046 (0.020); spherical equivalent = 0.387 (0.022) (Fig. 1). However, once again, these numerical differences in SNP-heritability estimated using dichotomous vs. continuous trait definitions were not large enough to attain statistical support. Dominance effects, investigated using GREMLd, were found to make a negligible contribution to the heritability of all three traits (Online Resource 3).

In light of these findings, we elected to model astigmatism as a continuous trait for our primary GWAS analyses, with follow-up sensitivity analyses using a mid-range case-definition threshold of 1.00 D, which is also the threshold most commonly adopted in the clinical literature.

### Genome-wide association studies (GWAS)

After restricting the analysis sample to individuals of White British-ancestry and applying exclusions for eye disorders with the potential to alter the level of astigmatism, there were 86,355 individuals available for inclusion in the GWAS for corneal astigmatism and 88,005 individuals in the GWAS for refractive astigmatism. After applying marker restrictions, there were 5,901,671 and 5,900,115 markers available for inclusion in the corneal and refractive astigmatism GWAS analyses, respectively.

**Fig. 2:**
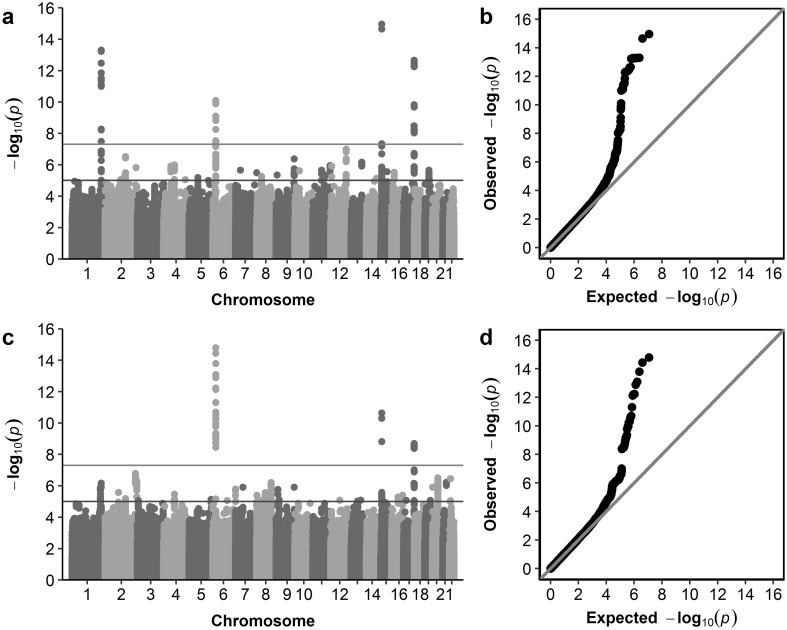
Manhattan and Quantile–Quantile plots for GWAS of corneal astigmatism and refractive astigmatism using BOLT-LMM. **a, b** Corneal astigmatism; **c** and **d** refractive astigmatism. Manhattan plots (**a, c**): upper horizontal line indicates the genome-wide significance threshold at *P* = 5 × 10^−8^; lower horizontal line indicates *P* = 1 × 10^−5^. Quantile–Quantile plots (**b, d**): *Y*-axis shows observed negative log_10_ *p* values and *X*-axis shows expected negative log_10_ *p* values according to the null hypothesis of no genetic association. Diagonal line = line of unity (observed = expected)

**Fig. 3:**
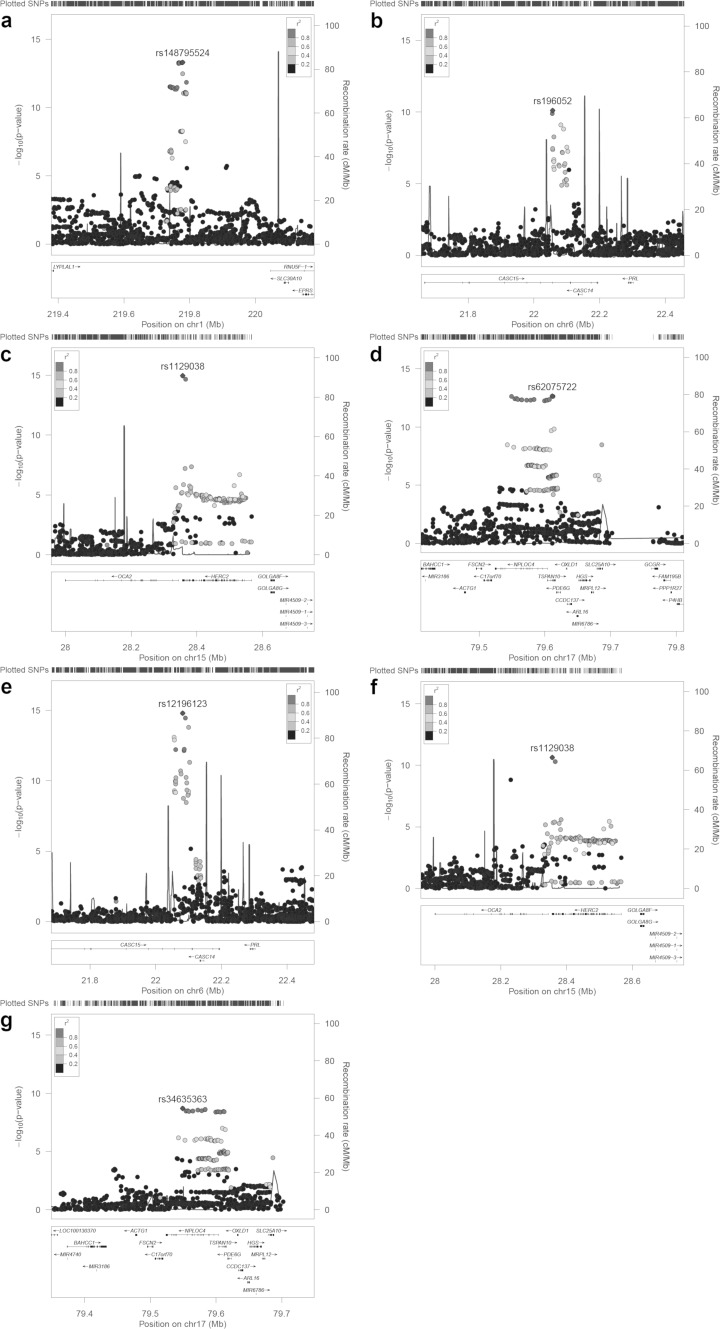
Regional association plots for loci demonstrating genome-wide significant association (*P* < 5 × 10^−8^) in GWAS for Corneal Astigmatism and Refractive Astigmatism using BOLT-LMM. **a**–**d** Corneal astigmatism; **e**–**g** refractive astigmatism. In order of chromosome: **a** rs12032649, **b** rs196052, **c** rs1129038, **d** rs62075722, **e** rs12196123, **f** rs1129038, and **g** rs34635363. Symbol shading denotes linkage disequilibrium (*r*^2^) values of variants with respect to the lead marker (named and highlighted). NB: rs14879552 is a synonym for rs12032649

For our primary analyses, we carried out single marker association tests using the mixed linear model approach implemented in BOLT-LMM, since this provides greater power than tests using standard linear regression (Yang et al. 2014). GWAS analyses identified 89 and 45 markers achieving genome-wide significant association (*P* < 5 × 10^−8^) for corneal astigmatism and refractive astigmatism, respectively (Online Resource 4). Specifically, for corneal astigmatism, genome-wide significant markers clustered in four regions (Figs. 2, 3), while for refractive astigmatism, they clustered in three regions (Figs. 2, 3).

For corneal astigmatism, the nearest gene at each of the four genome-wide significant loci was *ZC3H11B* (top marker: rs12032649, *P* = 5.00 × 10^−14^), *LINC00340* (top marker: rs196052, *P* = 7.80 × 10^−11^), *HERC2* (top marker: rs1129038, *P* = 1.10 × 10^−15^) and *TSPAN10*/*NPLOC4* (top marker: rs62075722, *P* = 2.20 × 10^−13^) (Table 1). None of these loci have previously shown genome-wide significant association with corneal or refractive astigmatism. At the only previously identified genome-wide significant locus for corneal astigmatism, the promoter region of the *PDGFRA* gene at 4q12, the marker demonstrating strongest association was rs4864857 (*P* = 1.20 × 10^−6^). Conditional analyses carried out by conditioning on the top marker at each of the four novel genome-wide significant loci suggested that these four association signals were each driven by a single causal marker (Online Resource 5). However, the strength of the association signal at the *HERC2* locus when conditioning on top marker rs1129038 did yield a suggestive association signal within the adjacent *OCA2* gene (top marker: rs1800407, *P* = 9.88 × 10^−6^).

**Table 1:** Markers achieving association test *P* values < 1 × 10^−5^ in GWAS for Corneal astigmatism analysed as a continuous trait with BOLT-LMM

Marker	Chromosome	Position	Effect allele	Other allele	EAF	HWE *P* value	Effect (SE)	*P* value	Nearest gene
rs1129038	15	28,356,859	C	T	0.215	0.621	− 0.028 (0.003)	1.10 × 10^−15^	*HERC2*
rs12032649	1	219,778,959	T	G	0.614	0.138	− 0.022 (0.003)	5.00 × 10^−14^	*ZC3H11B*
rs62075722	17	79,611,271	A	G	0.358	0.594	0.022 (0.003)	2.20 × 10^−13^	*TSPAN10*
rs196052	6	22,057,200	T	A	0.622	0.477	0.019 (0.003)	7.80 × 10^−11^	*LINC00340*
rs61935843	12	116,617,757	C	A	0.918	0.873	0.028 (0.005)	1.00 × 10^−7^	*MED13L*
rs1579050	2	153,364,527	A	G	0.425	0.017	0.015 (0.003)	3.00 × 10^−7^	*FMNL2*
rs10993820	9	136,707,730	A	G	0.791	0.803	− 0.018 (0.003)	4.20 × 10^−7^	*VAV2*
rs9517490	13	99,584,305	T	C	0.300	0.703	0.015 (0.003)	6.80 × 10^−7^	*DOCK9*
rs1353386	4	81,947,080	A	C	0.148	0.808	0.019 (0.004)	1.00 × 10^−6^	*BMP3*
rs7931326	11	130,276,347	C	G	0.935	0.716	− 0.028 (0.006)	1.10 × 10^−6^	*ADAMTS8*
rs4864857	4	55,089,814	T	C	0.784	0.166	− 0.017 (0.003)	1.20 × 10^−6^	*PGDFRA*
rs112947941	12	6,997,808	A	G	0.931	0.074	0.028 (0.006)	1.20 × 10^−6^	*DSTNP2*
rs12473604	2	232,401,893	G	A	0.775	0.258	− 0.016 (0.003)	1.50 × 10^−6^	*NMUR1*
rs35313216	11	66,224,195	G	A	0.928	0.304	0.026 (0.005)	2.20 × 10^−6^	*LOC100505524*
rs11084579	19	31,802,723	G	A	0.666	0.270	0.014 (0.003)	2.20 × 10^−6^	*TSHZ3*
rs10279904	7	36,806,587	C	T	0.987	0.363	− 0.058 (0.0012)	2.20 × 10^−6^	*AOAH*
rs138016380	10	34,449,466	C	A	0.976	0.821	− 0.044 (0.009)	2.40 × 10^−6^	*PARD3*
rs11639295	15	67,460,757	C	T	0.706	0.902	0.015 (0.003)	2.70 × 10^−6^	*SMAD3*
rs16971637	16	19,155,288	A	C	0.959	0.931	− 0.033 (0.007)	3.00 × 10^−6^	*ITPRIPL2*
rs117023057	7	158,823,501	G	A	0.984	0.092	− 0.054 (0.012)	3.20 × 10^−6^	*VIPR2*
rs2445565	11	86,803,194	G	C	0.470	0.119	− 0.013 (0.003)	4.20 × 10^−6^	*TMEM135*
rs12551905	9	7,760,772	T	C	0.985	0.908	− 0.054 (0.012)	4.50 × 10^−6^	*C9orf123*
rs149846728	8	36,770,379	G	A	0.949	0.332	0.030 (0.006)	5.40 × 10^−6^	*KCNU1*
rs62169220	2	145,225,071	A	G	0.850	0.354	− 0.018 (0.004)	5.80 × 10^−6^	*ZEB2*
rs830557	5	67,608,743	C	T	0.529	0.368	0.013 (0.003)	6.50 × 10^−6^	*PIK3R1*
rs56274409	14	96,690,828	A	T	0.944	0.567	− 0.028 (0.006)	7.20 × 10^−6^	*BDKRB2*
rs6741982	2	117,793,229	A	G	0.961	0.748	− 0.033 (0.007)	8.70 × 10^−6^	*MTND2P21*
rs6536686	4	163,731,498	C	T	0.192	0.921	0.016 (0.004)	8.80 × 10^−6^	*MIR4454*
rs57770499	19	36,260,996	G	A	0.820	0.808	− 0.016 (0.004)	1.00 × 10^−5^	*C19orf55*
rs13181991	5	146,163,470	C	T	0.917	0.327	0.023 (0.005)	1.00 × 10^−5^	*PPP2R2B*

*EAF* effect allele frequency, *HWE P value P* value from the Hardy–Weinberg disequilibrium test, *SE* standard error, *NB* markers within ± 500 kb of listed (lead) marker are not included in this list

As mentioned above, for refractive astigmatism, markers achieving genome-wide significant association clustered in three regions (Table 2): *LINC00340*, (top marker: rs12196123, *P* = 1.60 × 10^−15^), *HERC2* (top marker: rs1129038, *P* = 2.30 × 10^−11^) and *TSPAN10*/*NPLOC4* (top marker: rs34635363, *P* = 2.00 × 10^−9^). Notably, all of these loci also demonstrated significant association with corneal astigmatism. Conditional analyses for these genome-wide significant loci also suggested these association signals were each driven by a single causal marker with the exception of the association signal at *HERC2* which appeared to be driven by an additional independent causal marker within the *OCA2* gene at rs1800407 (*P* = 9.03 × 10^−15^) (Online Resource 6). Conditioning on both rs1129038 and rs1800407 at the *HERC2*-*OCA2* locus resulted in a suggestive association signal at rs7497044 (*P* = 1.90 × 10^−6^), an intronic variant within the nearby *GABRG3* gene. In a previous meta-analysis of GWAS for corneal curvature in European ancestry cohorts from Australia (Mishra et al. 2012), marker rs17137734 within *GABRG3* achieved suggestive association (*P* = 9 × 10^−6^). Pairwise LD between markers rs7497044, rs17137734, rs1129038 and rs1800407 varies from low to negligible in Europeans (*r*^2^ = 0.0–0.1).

**Table 2:** Markers achieving association test-*P* values < 1 × 10^−5^ in GWAS for refractive astigmatism analysed as a continuous trait with BOLT-LMM

Marker	Chromosome	Position	Effect allele	Reference allele	EAF	HWE *P* value	Effect (SE)	*P* value	Nearest gene
rs12196123	6	22,082,263	C	T	0.443	0.659	− 0.023 (0.003)	1.60 × 10^−15^	*LINC00340*
rs1129038	15	28,356,859	C	T	0.215	0.047	− 0.023 (0.004)	2.30 × 10^−11^	*HERC2*
rs34635363	17	79,549,250	G	A	0.641	0.759	− 0.018 (0.003)	2.00 × 10^−9^	*NPLOC4*
rs10177414	2	228,211,470	T	C	0.598	0.020	− 0.015 (0.003)	1.60 × 10^−7^	*MFF*
rs6029691	20	40,094,364	C	G	0.688	0.621	− 0.016 (0.003)	3.10 × 10^−7^	*CHD6*
rs139743	22	25,299,429	A	G	0.582	0.675	− 0.015 (0.003)	3.50 × 10^−7^	*SGSM1*
rs77008212	2	239,307,113	A	G	0.913	0.612	− 0.026 (0.005)	4.70 × 10^−7^	*TRAF3IP1*
rs141045115	21	42,387,103	G	T	0.971	0.795	− 0.043 (0.009)	6.00 × 10^−7^	*LINC00323*
rs10435539	8	109,167,551	G	A	0.795	0.051	0.018 (0.004)	6.10 × 10^−7^	*AURKBPS1*
rs116771750	1	219,699,050	T	C	0.965	0.589	− 0.039 (0.008)	6.70 × 10^−7^	*ZC3H11B*
rs79999086	20	1,058,226	T	C	0.980	0.245	− 0.051 (0.011)	1.20 × 10^−6^	*LCN1P2*
rs17172445	7	55,189,215	G	T	0.973	0.687	− 0.043 (0.009)	1.20 × 10^−6^	*EGFR*
rs11244084	9	136,191,010	C	T	0.926	0.684	− 0.027 (0.005)	1.20 × 10^−6^	*PSMF1*
rs115732928	1	214,154,088	A	T	0.952	0.115	− 0.033 (0.007)	1.30 × 10^−6^	*PROX1-AS1*
rs57717978	6	170,267,973	C	T	0.880	0.354	− 0.021 (0.004)	1.60 × 10^−6^	*LOC101929541*
rs10494951	1	212,429,259	G	A	0.806	0.347	0.018 (0.004)	1.60 × 10^−6^	*LINC00574*
rs141720143	9	13,237,186	A	C	0.987	0.349	− 0.060 (0.013)	1.70 × 10^−6^	*MPDZ*
rs77909168	2	100,555,866	C	G	0.985	0.872	− 0.056 (0.012)	2.60 × 10^−6^	*AFF3*
rs56288719	8	64,825,393	T	C	0.962	0.207	− 0.036 (0.008)	2.70 × 10^−6^	*LOC286184*
rs6535231	4	81,951,911	G	A	0.041	0.386	0.034 (0.007)	3.40 × 10^−6^	*BMP3*
rs192290664	16	82,799,444	A	G	0.978	0.410	− 0.046 (0.010)	3.90 × 10^−6^	*CDH13*
rs12547340	8	1,149,028	G	T	0.790	0.445	0.016 (0.004)	4.00 × 10^−6^	*DLGAP2*
rs149069109	16	48,972,368	C	T	0.977	0.920	− 0.044 (0.010)	5.20 × 10^−6^	*KLF8P1*
rs6434068	2	153,357,541	G	C	0.428	0.647	0.013 (0.003)	6.50 × 10^−6^	*FMNL2*
rs891933	5	167,591,402	C	T	0.537	0.329	0.013 (0.003)	7.30 × 10^−6^	*TENM2*
rs116945318	9	21,638,723	A	C	0.985	0.779	− 0.055 (0.012)	7.50 × 10^−6^	*KHSRPP1*
rs974420	13	93,152,458	G	A	0.621	0.012	0.013 (0.003)	8.10 × 10^−6^	*GPC5*
rs34314196	8	40,998,854	T	A	0.874	0.724	− 0.019 (0.004)	8.20 × 10^−6^	*SFRP1*
rs117949737	12	69,299,466	G	A	0.974	0.594	0.040 (0.009)	8.30 × 10^−6^	*CPM*
rs8104928	19	42,130,284	A	G	0.972	0.185	− 0.039 (0.009)	8.40 × 10^−6^	*CEACAM4*
rs75819168	17	19,651,119	C	G	0.984	0.814	− 0.051 (0.011)	8.50 × 10^−6^	*ALDH3A1*
rs117812342	6	108,473,446	A	C	0.976	0.766	− 0.042 (0.009)	8.60 × 10^−6^	*OSTM1-AS1*
rs55939894	3	7,963,288	A	G	0.813	0.845	− 0.016 (0.004)	8.70 × 10^−6^	*LOC101927394*
rs513910	13	69,095,822	T	C	0.726	0.995	0.014 (0.003)	9.10 × 10^−6^	*RPS3AP52*
rs62316885	4	75,558,798	C	T	0.962	0.879	− 0.033 (0.008)	1.00 × 10^−5^	*AREGB*

*EAF* effect allele frequency, *HWE P value P* value from the Hardy–Weinberg disequilibrium test, *SE* standard error, *NB* markers within ± 500 kb of listed (lead) marker are not included in this list

Genomic inflation factors (*λ*_GC_) were 1.094 for corneal astigmatism and 1.045 for refractive astigmatism; however, when accounting for the polygenic nature of these respective traits using the intercepts from LD Score regression (*λ*_LDSC_), inflation due to uncorrected population effects was estimated to be considerably lower for both traits (*λ*_LDSC_ = 1.023 and 1.005 for corneal and refractive astigmatism, respectively).

As validation for the use of mixed linear models to conduct the association tests, analyses were repeated using linear regression (implemented in PLINK 2.0) and an identical set of covariates (note that this approach necessitated the analysis of a smaller sample of *unrelated* individuals). For corneal astigmatism, all four loci identified using the mixed linear model analysis also demonstrated genome-wide significant association using the linear regression model in PLINK, while only two of the three loci originally associated with refractive astigmatism (*LINC00340* and *HERC2*) continued to demonstrate genome-wide significant association (Online Resources 7, 8, 11a, 11c, 12a and 12c). Additional sensitivity analyses were performed using logistic regression for the same groups of individuals and covariates as analysed by linear regression, with cases defined as individuals with corneal or refractive astigmatism ≥ 1.00 D. Here, three of the four previously identified loci, near the genes *ZC3H11B, HERC2* and *TSPAN10*/*NPLOC4*, demonstrated genome-wide significant association for corneal astigmatism, while only the *LINC00340* locus continued to demonstrate genome-wide significant association for refractive astigmatism (Online Resources 9, 10, 11b, 11d, 12b and 12d). In all instances, the association signals were reduced using linear and logistic regression compared to the mixed linear model analyses. This was likely due to the substantial drop in sample size necessitated by standard regression-based methods, which cannot account for relatedness between individuals. Tests for a difference in genetic effect size in male vs. female participants for the genome-wide significant loci for corneal astigmatism and refractive astigmatism did not identify any such differences after accounting for multiple testing (Online Resources 13 and 14), suggesting that the newly identified variants confer susceptibility to astigmatism in both sexes.

### Gene-based and gene-set tests

To identify potential candidate genes and biological mechanisms enriched with markers attaining low but not necessarily genome-wide significant *P* values from GWAS, gene-based and gene-set tests were performed in MAGMA using the results of the mixed linear model analyses. The gene-based analysis for corneal astigmatism identified 37 genes at a false discovery rate (FDR) < 0.05. These genes included a cluster of nine genes at the *TSPAN10*/*NPLOC4* locus (17q25.3; FDR = 2.10 × 10^−6^) along with the genes *HERC2* (15q13.1; FDR = 2.30 × 10^−4^), *PDGFRA* (4q12; FDR = 5.21 × 10^−4^), and *B3GNT7* (2q37.1; FDR = 3.66 × 10^−3^) (Online Resource 15). For refractive astigmatism, gene-based analysis identified 35 genes with FDR < 0.05. Of these genes, seven were clustered at the gene-dense *TSPAN10*/*NPLOC4* locus (17q25.3; FDR = 5.00 × 10^−3^). Additional genes identified included *TMEM211* (22q11.23; FDR = 4.96 × 10^−3^), *PROX1* (1q32.3; FDR = 4.96 × 10^−3^), *HERC2* (15q13.1; FDR = 5.00 × 10^−3^), and *PLAUR* (19q13.31; FDR = 5.69 × 10^−3^) (Online Resource 16). Gene-set analyses in MAGMA yielded non-significant findings after correction for multiple testing.

### SNP-heritability and genetic correlation analyses using GWAS summary statistics

LD Score regression-based SNP-heritability estimates for corneal and refractive astigmatism, calculated from the GWAS summary statistics for the continuous trait analyses described above, were 0.036 (SE = 0.006, *P* = 4.34 × 10^−10^) and 0.034 (SE = 0.006, *P* = 2.71 × 10^−9^), respectively (Table 3). These estimates were lower—albeit with overlapping 95% confidence intervals—compared to the equivalent estimates obtained directly using GCTA (Table 3).

**Table 3:** Estimates of SNP-heritability (*h*^2^_SNP_) using GCTA and LSDC

Trait	Method	No. of markers	Sample size	*h* ^2^_SNP_	SE	*P* value
Corneal astigmatism	GCTA	732,404	27,707	0.061	0.021	1.19 × 10^−3^
	LDSC	864,048	86,355	0.036	0.006	4.34 × 10^−10^
Refractive astigmatism	GCTA	732,404	28,378	0.046	0.020	7.74 × 10^−3^
	LDSC	863,851	88,005	0.034	0.006	2.71 × 10^−9^

*h* ^2^_SNP_ SNP-heritability, *SE* standard error, *P value* test of the null hypothesis (*h*^2^_SNP_ = 0)

The phenotypic correlation between each pair of the three refractive traits (corneal astigmatism, refractive astigmatism and spherical equivalent) were all significantly different from the null hypothesis of zero (Table 4). The genetic correlation between corneal and refractive astigmatism, calculated using LD Score regression, was 0.85 (SE = 0.068, *P* = 1.37 × 10^−35^). In contrast, genetic correlations between the astigmatism traits and spherical equivalent were weaker at −0.108 for corneal astigmatism and spherical equivalent; and − 0.104 for refractive astigmatism and spherical equivalent (Table 4). In both of the latter instances, the negative sign of the genetic correlation is due to the signs of the respective traits: astigmatism is always positive, whereas spherical equivalent values are negative for myopic individuals. Additionally, these genetic correlations were not significantly different from zero (*P* = 0.067 and 0.071, respectively; Table 4).

**Table 4:** Genetic and phenotypic correlations between pairs of refractive error traits

Trait pairs	No. of variants	Genetic correlation (SE)	*P* value	Phenotypic correlation	95% CI
CA and RA	862,521	0.851 (0.068)	1.37 × 10^−35^	0.615	0.610 to 0.620
CA and MSE	862,524	− 0.108 (0.059)	0.067	− 0.093	− 0.100 to − 0.085
RA and MSE	863,831	− 0.104 (0.057)	0.071	− 0.156	− 0.163 to − 0.148

Genotypic correlations were obtained using LDSC and summary statistics from BOLT-LMM analyses (*N* = 86,335 or 88,005). All phenotypic correlations are Pearson correlations for 63,466 unrelated individuals included in GWAS for these respective traits and with data available for all three refractive error measures

*P* values refer to the null hypothesis of zero genetic correlation between traits

*CA* corneal astigmatism, *RA* refractive astigmatism, *MSE* mean spherical equivalent

## Discussion

The GWAS analyses undertaken here for corneal and refractive astigmatism are the largest performed to date, and led to the discovery of four novel genome-wide significant loci associated with corneal astigmatism, and two novel genome-wide significant loci associated with refractive astigmatism.

The SNP-heritability estimates for corneal and refractive astigmatism calculated here were much lower than previous broad-sense and narrow-sense heritability estimates from twin and family studies (approximately 5% vs. 50%), with a negligible contribution of dominance effects. The SNP-heritability estimates for spherical equivalent calculated here were approximately 50% of those obtained from twin studies (Hammond et al. 2001; Dirani et al. 2006). It should be noted that heritability estimation in twin and family studies takes into consideration a wider range of sources of genetic variation, such as the effects of rare variants and dominance/epistatic genetic effects; whereas SNP-heritability estimates only take account of the additive contribution of commonly occurring variants. It should also be noted that both of the methods we used to estimate SNP-heritability (GCTA-GREML and LD score regression) make the assumption that the effect sizes of all causal variants conform to a single Gaussian distribution, which implies that effect sizes are independent of MAF and local LD (Evans et al. 2018). Across 19 traits, Speed et al. (2017) found that departure from this assumption led to an underestimation of SNP-heritability by approximately 40%. Nevertheless, the very marked reduction in SNP-heritability for the astigmatism traits compared to spherical equivalent suggests either a major role for rare variants in the development of astigmatism and/or that previous heritability estimates were biased upwards due to misallocation of environmental effects as genetic effects. With regard to spherical equivalent, the SNP-heritability estimate obtained here (*h*^2^_SNP_ = 0.387; *P* < 1 × 10^−10^) was similar to a previously published estimate, which suggested a SNP-heritability of 0.35 (Guggenheim et al. 2015). Importantly, the sample used to generate this previous estimate consisted of children aged 7–15 years-old, whereas the current investigation utilised a sample of older adults (40–69 years old) and heritability estimates are sensitive to population demographics such as age and ethnicity (Visscher et al. 2008). To date, no additional estimates of SNP-heritability for spherical equivalent, myopia or astigmatism have been reported in the published literature with the exception of conference abstracts (Miyake et al. 2013; Hysi et al. 2014).

It could be argued that astigmatism and spherical equivalent refractive error share little genetic susceptibility since the genetic correlations between these respective pairs of traits were not significantly different from zero. Possible explanations for these relatively weak genetic correlations are the differences in their respective SNP-heritabilities, the number of genome-wide significant associations identified for each trait, and the fact that spherical equivalent was included as a covariate in our GWAS analyses for astigmatism. Few commonly occurring markers have demonstrated association with astigmatism, yet it is notable that the novel loci for corneal and refractive astigmatism identified in our GWAS analyses have previously shown association with other ocular traits. Table 5 contains a summary of the loci achieving genome-wide significant association in our investigation and previously identified associations of these loci with other ocular traits, as reported in the NHGRI-EBI catalogue of published genome-wide association studies (MacArthur et al. 2017). With the exception of the association signal at *HERC2*/*OCA2*, the majority of the astigmatism susceptibility loci have demonstrated association with spherical equivalent-related traits in previous GWAS analyses. Furthermore, despite the overlap in genetic variants associated with spherical refractive error and astigmatism, it is noteworthy that the variants most strongly associated with each condition appear to be distinct (for example, the variants consistently found to be most strongly associated with spherical refractive error—namely, those at the *GJD2* and *LAMA2* loci—were not amongst the loci most strongly associated with corneal astigmatism and refractive astigmatism). Therefore, the common variants that confer susceptibility to astigmatism appear to comprise only a subset of the wider collection of common genetic variants contributing to susceptibility to spherical equivalent refractive error.

**Table 5:** Previously observed associations with ocular traits at the newly identified susceptibility loci for corneal astigmatism

Gene	Region	Previous associations	References
*ZC3H11B*	1q41	Axial length Pathological myopia	Cheng et al. (2013) Fan et al. (2012)
*LINC00340*	6p22.3	Refractive astigmatism Spherical equivalent	Li et al. (2015) Fan et al. (2016)
*HERC2*	15q13.1	Eye colour	Kayser et al. (2008)
*TSPAN10*/*NPLOC4*	17q25.3	Myopia Advanced age-related macular degeneration Eye colour	Pickrell et al. (2016) Fritsche et al. (2016) Liu et al. (2010)

Comparison of the genotypic correlations calculated here for UK Biobank participants against those reported previously by the CREAM consortium (Shah et al. 2018), revealed limited similarity. Specifically, in the UK Biobank sample, the genetic correlation between corneal and refractive astigmatism was higher (0.851 vs. 0.233), that between corneal astigmatism and spherical equivalent was similar (− 0.108 vs − 0.024) and that between refractive astigmatism and spherical equivalent was lower (− 0.104 vs. 0.773) than in the CREAM meta-analysis sample (Shah et al. 2018). Potential reasons for the lack of concordance were that the genetic correlations from the CREAM study were based on analyses of corneal and refractive astigmatism considered as dichotomous traits rather than continuous traits, the smaller sample size of the CREAM study, and the inclusion of participants with a wider range of ages and ethnic backgrounds by CREAM. These methodological differences resulted in much less precise SNP-heritability and genetic correlation estimates in the CREAM study than were obtained here (the standard errors here were 5–10-fold lower than those in the CREAM study). Accordingly, the more precise results presented here (Table 4) are likely to represent a more accurate representation of the true genetic correlations.

Markers near the protein coding gene *ZC3H11B* (zinc finger CCCH-type containing 11B) on chromosome 1 (1q41) have previously demonstrated association with pathological (high) myopia in Asian ancestry cohorts and with axial length in both European and Asian ancestry individuals (Cheng et al. 2013; Fan et al. 2012). Ocular expression of *ZC3H11B* has been identified in human retinal and scleral tissues (Fan et al. 2012). *LINC00340*, also known as *CASC15* (cancer susceptibility 15), is a long, non-coding RNA transcript located on chromosome 6 (6p22.3). In a previous meta-analysis of GWAS from European and Asian ancestry cohorts, this locus demonstrated genome-wide significant association with spherical equivalent refractive error (Fan et al. 2016) and suggestive association (*P* < 1 × 10^−5^) with refractive astigmatism (Li et al. 2015). For both studies, associations at the locus appear to be largely driven by signals from European-ancestry cohorts, with little association demonstrated by their Asian ancestry counterparts. The protein coding gene *HERC2* (HECT and RLD domain containing E3 ubiquitin protein ligase 2) and its neighbouring gene *OCA2* (Oculocutaneous albinism type 2) on chromosome 15 (15q13.1) have both previously demonstrated association with eye, skin and hair pigmentation (Kayser et al. 2008; Sturm and Larsson 2009; Liu et al. 2010). *TSPAN10* (Tetraspanin 10), also known as Oculospanin, is a protein-coding gene located within a gene-dense region on chromosome 17 (17q25.3). This gene regulates the transmembrane metalloprotease *ADAM10* as part of the Notch signalling pathway (Charrin et al. 2014). Ocular expression of *TSPAN10* has been identified in the iris, ciliary body and retinal pigment epithelium (Wistow et al. 2002) and this locus has previously demonstrated genome-wide significant association with eye colour, myopia and age-related macular degeneration (Pickrell et al. 2016; Fritsche et al. 2016; Liu et al. 2010). The identification of 3 genes (*HERC2, OCA2* and *TSPAN10*) associated with eye colour and astigmatism implies that certain eye colour(s) may confer susceptibility to astigmatism or that these eye colour-related genes have distinct, pleiotropic actions that lead to astigmatism. (While it is possible that astigmatism confers susceptibility to certain eye colours, or that susceptibility to both eye colour and astigmatism is mediated via an intermediate genetically determined trait, we consider these latter options less likely). It is notable that Pan et al. (2018) recently identified an association between iris colour (dark vs. light brown) and spherical equivalent refractive error in a sample of Chinese school children. Hence, the relationship between eye colour and refractive errors may be a promising avenue for further research.

Our primary GWAS analyses were conducted using mixed linear models as implemented in BOLT-LMM (Loh et al. 2015, 2018). Mixed linear models have the advantage over standard linear regression that they can correct for residual population stratification and relatedness within the study sample, which can otherwise lead to reduced power or an excess of false positive association signals (Yang et al. 2014). Due to the increased sample size the mixed linear model approach allows, the genome-wide significant association signals obtained here were stronger than those obtained from standard linear regression. An important limitation of using mixed linear models for association studies is that they can produce unreliable results for dichotomous traits (Chen et al. 2016; Yang et al. 2014); hence, we only considered corneal and refractive astigmatism as continuous traits for the mixed model analyses. Nonetheless, the results were similar to those obtained using PLINK 2.0 with corneal and refractive astigmatism considered as continuous traits and as dichotomous traits defined using a threshold of 1.00 D for assigning case status (Online Resources 7–12).

It should be noted that the magnitudes of corneal and refractive astigmatism in this UK Biobank sample follow a similar change with age as observed in other European ancestry samples (Sanfilippo et al. 2015; Schuster et al. 2017), with corneal astigmatism relatively stable with increasing age and refractive astigmatism gradually increasing with age (Online Resource 1). Whilst these changes with age could influence the ability to detect genetic variants associated with these astigmatism traits, this was mitigated against by the inclusion of age as a quantitative covariate in all analyses. However, there may be some residual effects not accounted for.

As increasing magnitudes of astigmatism are correlated with increasing magnitudes of spherical refractive error (Guggenheim and Farbrother 2004; Kronfeld and Devney 1930), spherical equivalent was included as a covariate to negate the effects of this correlation as a potential driver of association signals. Using spherical equivalent is more conservative an adjustment than using the spherical refractive component only, as this also adjusts for the contribution of refractive astigmatism to the overall refractive error of the individual (Guggenheim and Farbrother 2004). Further sensitivity analyses, in which GWAS analyses were repeated without including spherical equivalent as a covariate, did not appreciably alter the findings.

In summary, we have conducted the largest genome-wide association studies for corneal and refractive astigmatism to date and identified four novel loci for corneal astigmatism, two of which are also novel loci for refractive astigmatism. It was notable that all of these novel loci have previously been associated with different ocular traits (Table 5), most prominently spherical equivalent refractive error. However, the astigmatism association signals were genome-wide significant even after adjusting for the effects of spherical equivalent, confirming that they represent independent associations, thus lending further support to the concept of shared genetic susceptibility for myopia and astigmatism.

## Electronic supplementary material

Below is the link to the electronic supplementary material.

Supplementary material 1 (PDF 1308 KB)

## References

[CR1] Abraham G, Inouye M (2014). Fast principal component analysis of large-scale genome-wide data. PLoS One.

[CR2] Allen NE, Sudlow C, Peakman T, Collins R, Biobank UK (2014). UK biobank data: come and get it. Sci Transl Med.

[CR3] Brodie A, Azaria JR, Ofran Y (2016). How far from the SNP may the causative genes be?. Nucleic Acids Res.

[CR4] Bulik-Sullivan B, Finucane HK, Anttila V, Gusev A, Day FR, Loh PR, ReproGen C, Psychiatric Genomics C (2015). Genetic Consortium for Anorexia Nervosa of the Wellcome Trust Case Control C et al. An atlas of genetic correlations across human diseases and traits. Nat Genet.

[CR5] Bulik-Sullivan BK, Loh PR, Finucane HK, Ripke S, Yang J, Genomics C, Patterson N, Daly MJ, Price AL, Schizophrenia Working Group of the Psychiatric (2015). LD Score regression distinguishes confounding from polygenicity in genome-wide association studies. Nat Genet.

[CR6] Bycroft C, Freeman C, Petkova D, Band G, Elliott LT, Sharp K, Motyer A, Vukcevic D, Delaneau O (2017). Genome-wide genetic data on ~ 500,000 UK Biobank participants. bioRxiv.

[CR7] Chang CC, Chow CC, Tellier LC, Vattikuti S, Purcell SM, Lee JJ (2015). Second-generation PLINK: rising to the challenge of larger and richer datasets. Gigascience.

[CR8] Charrin S, Jouannet S, Boucheix C, Rubinstein E (2014). Tetraspanins at a glance. J Cell Sci.

[CR9] Chen H, Wang C, Conomos MP, Stilp AM, Li Z, Sofer T, Szpiro AA, Chen W, Brehm JM (2016). Control for population structure and relatedness for binary traits in genetic association studies via logistic mixed models. Am J Hum Genet.

[CR10] Cheng CY, Schache M, Ikram MK, Young TL, Guggenheim JA, Vitart V, MacGregor S, Verhoeven VJ, Barathi VA (2013). Nine loci for ocular axial length identified through genome-wide association studies, including shared loci with refractive error. Am J Hum Genet.

[CR11] Clementi M, Angi M, Forabosco P, Di Gianantonio E, Tenconi R (1998). Inheritance of astigmatism: evidence for a major autosomal dominant locus. Am J Hum Genet.

[CR12] Corradin O, Cohen AJ, Luppino JM, Bayles IM, Schumacher FR, Scacheri PC (2016). Modeling disease risk through analysis of physical interactions between genetic variants within chromatin regulatory circuitry. Nat Genet.

[CR13] Corvin A, Craddock N, Sullivan PF (2010). Genome-wide association studies: a primer. Psychol Med.

[CR14] Cumberland PM, Bao Y, Hysi PG, Foster PJ, Hammond CJ, Rahi JS, UK Biobank Eyes & Vision Consortium (2015). Frequency and distribution of refractive error in adult life: methodology and findings of the UK biobank study. PLoS One.

[CR15] Davies G, Marioni RE, Liewald DC, Hill WD, Hagenaars SP, Harris SE, Ritchie SJ, Luciano M, Fawns-Ritchie C (2016). Genome-wide association study of cognitive functions and educational attainment in UK Biobank (N = 112 151). Mol Psychiatry.

[CR16] de Leeuw CA, Mooij JM, Heskes T, Posthuma D (2015). MAGMA: generalized gene-set analysis of GWAS data. PLoS Comput Biol.

[CR17] de Leeuw CA, Neale BM, Heskes T, Posthuma D (2016). The statistical properties of gene-set analysis. Nat Rev Genet.

[CR18] Devlin B, Roeder K, Wasserman L (2001). Genomic control, a new approach to genetic-based association studies. Theor Popul Biol.

[CR19] Dirani M, Chamberlain M, Shekar SN, Islam AF, Garoufalis P, Chen CY, Guymer RH, Baird PN (2006). Heritability of refractive error and ocular biometrics: the Genes in Myopia (GEM) twin study. Invest Ophthalmol Vis Sci.

[CR20] Dirani M, Chan YH, Gazzard G, Hornbeak DM, Leo SW, Selvaraj P, Zhou B, Young TL, Mitchell P (2010). Prevalence of refractive error in Singaporean Chinese children: the strabismus, amblyopia, and refractive error in young Singaporean Children (STARS) study. Invest Ophthalmol Vis Sci.

[CR21] Evans LM, Tahmasbi R, Vrieze SI, Abecasis GR, Das S, Gazal S, Bjelland DW, de Candia TR, Goddard ME (2018). Comparison of methods that use whole genome data to estimate the heritability and genetic architecture of complex traits. Nat Genet.

[CR22] Fan Q, Zhou X, Khor CC, Cheng CY, Goh LK, Sim X, Tay WT, Li YJ, Ong RT (2011). Genome-wide meta-analysis of five Asian cohorts identifies PDGFRA as a susceptibility locus for corneal astigmatism. Plos Genet.

[CR23] Fan Q, Barathi VA, Cheng CY, Zhou X, Meguro A, Nakata I, Khor CC, Goh LK, Li YJ (2012). Genetic variants on chromosome 1q41 influence ocular axial length and high myopia. Plos Genet.

[CR24] Fan Q, Verhoeven VJ, Wojciechowski R, Barathi VA, Hysi PG, Guggenheim JA, Hohn R, Vitart V, Khawaja AP (2016). Meta-analysis of gene-environment-wide association scans accounting for education level identifies additional loci for refractive error. Nat Commun.

[CR25] Fritsche LG, Igl W, Bailey JN, Grassmann F, Sengupta S, Bragg-Gresham JL, Burdon KP, Hebbring SJ, Wen C (2016). A large genome-wide association study of age-related macular degeneration highlights contributions of rare and common variants. Nat Genet.

[CR26] Grjibovski AM, Magnus P, Midelfart A, Harris JR (2006). Epidemiology and heritability of astigmatism in Norwegian twins: an analysis of self-reported data. Ophthal Epidemiol.

[CR27] Guggenheim JA, Farbrother JE (2004). The association between spherical and cylindrical component powers. Optom Vis Sci.

[CR28] Guggenheim JA, McMahon G, Kemp JP, Akhtar S, St Pourcain B, Northstone K, Ring SM, Evans DM, Smith GD (2013). A genome-wide association study for corneal curvature identifies the platelet-derived growth factor receptor alpha gene as a quantitative trait locus for eye size in white Europeans. Mol Vis.

[CR29] Guggenheim JA, St Pourcain B, McMahon G, Timpson NJ, Evans DM, Williams C (2015). Assumption-free estimation of the genetic contribution to refractive error across childhood. Mol Vis.

[CR30] Guo Y, Jamison DC (2005). The distribution of SNPs in human gene regulatory regions. BMC Genom.

[CR31] Hammond CJ, Snieder H, Gilbert CE, Spector TD (2001). Genes and environment in refractive error: the twin eye study. Invest Ophthalmol Vis Sci.

[CR32] Harvey EM (2009). Development and treatment of astigmatism-related amblyopia. Optom Vis Sci.

[CR33] He M, Zeng J, Liu Y, Xu J, Pokharel GP, Ellwein LB (2004). Refractive error and visual impairment in urban children in southern china. Invest Ophthalmol Vis Sci.

[CR34] Howie B, Marchini J, Stephens M (2011). Genotype imputation with thousands of genomes. G3.

[CR35] Huynh SC, Kifley A, Rose KA, Morgan IG, Mitchell P (2007). Astigmatism in 12-year-old Australian children: comparisons with a 6-year-old population. Invest Ophthalmol Vis Sci.

[CR36] Hysi PG, Mangino M, Nag A, Williams KM, Hammond CJ (2014). High genomic coverage through NGS increases refractive error phenotypic variance explained by genes. Invest Ophthalmol Vis Sci.

[CR37] Kayser M, Liu F, Janssens AC, Rivadeneira F, Lao O, van Duijn K, Vermeulen M, Arp P, Jhamai MM (2008). Three genome-wide association studies and a linkage analysis identify HERC2 as a human iris color gene. Am J Hum Genet.

[CR38] Kiefer AK, Tung JY, Do CB, Hinds DA, Mountain JL, Francke U, Eriksson N (2013). Genome-wide analysis points to roles for extracellular matrix remodeling, the visual cycle, and neuronal development in myopia. Plos Genet.

[CR39] Kim MH, Zhao D, Kim W, Lim DH, Song YM, Guallar E, Cho J, Sung J, Chung ES (2013). Heritability of myopia and ocular biometrics in Koreans: the healthy twin study. Invest Ophthalmol Vis Sci.

[CR40] Koran ME, Thornton-Wells TA, Jahanshad N, Glahn DC, Thompson PM, Blangero J, Nichols TE, Kochunov P, Landman BA (2014). Impact of family structure and common environment on heritability estimation for neuroimaging genetics studies using sequential oligogenic linkage analysis routines. J Med Imaging.

[CR41] Kronfeld PC, Devney C (1930). The frequency of astigmatism. Arch Ophthalmol.

[CR42] Lee SH, Wray NR, Goddard ME, Visscher PM (2011). Estimating missing heritability for disease from genome-wide association studies. Am J Hum Genet.

[CR43] Li Q, Wojciechowski R, Simpson CL, Hysi PG, Verhoeven VJ, Ikram MK, Hohn R, Vitart V, Hewitt AW (2015). Genome-wide association study for refractive astigmatism reveals genetic co-determination with spherical equivalent refractive error: the CREAM consortium. Hum Genet.

[CR44] Liu F, Wollstein A, Hysi PG, Ankra-Badu GA, Spector TD, Park D, Zhu G, Larsson M, Duffy DL (2010). Digital quantification of human eye color highlights genetic association of three new loci. Plos Genet.

[CR45] Loh PR, Tucker G, Bulik-Sullivan BK, Vilhjalmsson BJ, Finucane HK, Salem RM, Chasman DI, Ridker PM, Neale BM (2015). Efficient Bayesian mixed-model analysis increases association power in large cohorts. Nat Genet.

[CR46] Loh P-R, Kichaev G, Gazal S, Schoech AP, Price AL (2018). Mixed model association for biobank-scale data sets. Nat Genet.

[CR47] Lopes MC, Hysi PG, Verhoeven VJ, Macgregor S, Hewitt AW, Montgomery GW, Cumberland P, Vingerling JR, Young TL (2013). Identification of a candidate gene for astigmatism. Invest Ophthalmol Vis Sci.

[CR48] MacArthur J, Bowler E, Cerezo M, Gil L, Hall P, Hastings E, Junkins H, McMahon A, Milano A (2017). The new NHGRI-EBI Catalog of published genome-wide association studies (GWAS Catalog). Nucleic Acids Res.

[CR49] McCarthy S, Das S, Kretzschmar W, Delaneau O, Wood AR, Teumer A, Kang HM, Fuchsberger C, Danecek P (2016). A reference panel of 64,976 haplotypes for genotype imputation. Nat Genet.

[CR50] Mishra A, Yazar S, Hewitt AW, Mountain JA, Ang W, Pennell CE, Martin NG, Montgomery GW, Hammond CJ (2012). Genetic variants near PDGFRA are associated with corneal curvature in Australians. Invest Ophthalmol Vis Sci.

[CR51] Miyake M, Yamashiro K, Nakanishi H, Nakata I, Akagi-Kurashige Y, Kumagai K, Tsujikawa A, Yamada R, Matsuda F (2013). Heritability estimation of axial length and refractive error explained by genome-wide single nucleotide polymorphisms. Invest Ophthalmol Vis Sci.

[CR52] Morgan PB (2007). Trends in UK contact lens prescribing. Optician.

[CR53] Pan CW, Qiu QX, Qian DJ, Hu DN, Li J, Saw SM, Zhong H (2018). Iris colour in relation to myopia among Chinese school-aged children. Ophthalmic Physiol Opt.

[CR54] Pickrell JK, Berisa T, Liu JZ, Segurel L, Tung JY, Hinds DA (2016). Detection and interpretation of shared genetic influences on 42 human traits. Nat Genet.

[CR55] Price AL, Patterson NJ, Plenge RM, Weinblatt ME, Shadick NA, Reich D (2006). Principal components analysis corrects for stratification in genome-wide association studies. Nat Genet.

[CR56] Pruim RJ, Welch RP, Sanna S, Teslovich TM, Chines PS, Gliedt TP, Boehnke M, Abecasis GR, Willer CJ (2010). LocusZoom: regional visualization of genome-wide association scan results. Bioinformatics.

[CR57] Purcell S, Neale B, Todd-Brown K, Thomas L, Ferreira MA, Bender D, Maller J, Sklar P, de Bakker PI (2007). PLINK: a tool set for whole-genome association and population-based linkage analyses. Am J Hum Genet.

[CR58] Quek TP, Chua CG, Chong CS, Chong JH, Hey HW, Lee J, Lim YF, Saw SM (2004). Prevalence of refractive errors in teenage high school students in Singapore. Ophthalmic Physiol Opt.

[CR59] Read SA, Collins MJ, Carney LG (2007). A review of astigmatism and its possible genesis. Clin Exp Optom.

[CR60] Sanfilippo PG, Yazar S, Kearns L, Sherwin JC, Hewitt AW, Mackey DA (2015). Distribution of astigmatism as a function of age in an Australian population. Acta Ophthalmol.

[CR61] Schork AJ, Thompson WK, Pham P, Torkamani A, Roddey JC, Sullivan PF, Kelsoe JR, O’Donovan MC, Furberg H (2013). All SNPs are not created equal: genome-wide association studies reveal a consistent pattern of enrichment among functionally annotated SNPs. Plos Genet.

[CR62] Schulze TG, McMahon FJ (2004). Defining the phenotype in human genetic studies: forward genetics and reverse phenotyping. Hum Hered.

[CR63] Schuster AK, Pfeiffer N, Schulz A, Hoehn R, Ponto KA, Wild PS, Blettner M, Beutel ME, Lackner KJ (2017). Refractive, corneal and ocular residual astigmatism: distribution in a German population and age-dependency—the Gutenberg health study. Graefes Arch Clin Exp Ophthalmol.

[CR64] Shah RL, Li Q, Zhao W, Tedja MS, Tideman JWL, Khawaja AP, Fan Q, Yazar S, Williams KM (2018). A genome-wide association study of corneal astigmatism: the CREAM consortium. Mol Vis.

[CR65] Speed D, Cai N, the UC, Johnson MR, Nejentsev S, Balding DJ (2017). Reevaluation of SNP heritability in complex human traits. Nat Genet.

[CR66] Sturm RA, Larsson M (2009). Genetics of human iris colour and patterns. Pigment Cell Melanoma Res.

[CR67] Subramanian A, Tamayo P, Mootha VK, Mukherjee S, Ebert BL, Gillette MA, Paulovich A, Pomeroy SL, Golub TR (2005). Gene set enrichment analysis: a knowledge-based approach for interpreting genome-wide expression profiles. Proc Natl Acad Sci USA.

[CR68] Sudlow C, Gallacher J, Allen N, Beral V, Burton P, Danesh J, Downey P, Elliott P, Green J (2015). UK biobank: an open access resource for identifying the causes of a wide range of complex diseases of middle and old age. PLoS Med.

[CR69] Tedja MS, Wojciechowski R, Hysi PG, Eriksson N, Furlotte NA, Verhoeven VJM, Iglesias AI, Meester-Smoor MA, Tompson SW (2018). Genome-wide association meta-analysis highlights light-induced signaling as a driver for refractive error. Nat Genet.

[CR70] Walter K, Min JL, Huang J, Crooks L, Memari Y, McCarthy S, Perry JR, Xu C, The UK 10K Consortium (2015). The UK10K project identifies rare variants in health and disease. Nature.

[CR71] Verhoeven VJ, Hysi PG, Wojciechowski R, Fan Q, Guggenheim JA, Hohn R, MacGregor S, Hewitt AW, Nag A (2013). Genome-wide meta-analyses of multiancestry cohorts identify multiple new susceptibility loci for refractive error and myopia. Nat Genet.

[CR72] Visscher PM, Hill WG, Wray NR (2008). Heritability in the genomics era—concepts and misconceptions. Nat Rev Genet.

[CR73] Vitale S, Ellwein L, Cotch MF, Ferris FL, Sperduto R (2008). Prevalence of refractive error in the United States, 1999–2004. Arch Ophthalmol.

[CR74] Wain LV, Shrine N, Miller S, Jackson VE, Ntalla I, Artigas MS, Billington CK, Kheirallah AK, Allen R (2015). Novel insights into the genetics of smoking behaviour, lung function, and chronic obstructive pulmonary disease (UK BiLEVE): a genetic association study in UK Biobank. Lancet Resp Med.

[CR75] Winkler TW, Kutalik Z, Gorski M, Lottaz C, Kronenberg F, Heid IM (2014). EasyStrata: evaluation and visualization of stratified genome-wide association meta-analysis data. Bioinformatics.

[CR76] Wistow G, Bernstein SL, Wyatt MK, Fariss RN, Behal A, Touchman JW, Bouffard G, Smith D, Peterson K (2002). Expressed sequence tag analysis of human RPE/choroid for the NEIBank Project: over 6000 non-redundant transcripts, novel genes and splice variants. Mol Vis.

[CR77] Yang J, Lee SH, Goddard ME, Visscher PM (2011). GCTA: a tool for genome-wide complex trait analysis. Am J Hum Genet.

[CR78] Yang J, Manolio TA, Pasquale LR, Boerwinkle E, Caporaso N, Cunningham JM, de Andrade M, Feenstra B, Feingold E (2011). Genome partitioning of genetic variation for complex traits using common SNPs. Nat Genet.

[CR79] Yang J, Ferreira T, Morris AP, Medland SE, Replication DIG, Meta-analysis C, Madden PA, Heath AC (2012). Genetic Investigation of ATC. Conditional and joint multiple-SNP analysis of GWAS summary statistics identifies additional variants influencing complex traits. Nat Genet.

[CR80] Yang J, Zaitlen NA, Goddard ME, Visscher PM, Price AL (2014). Advantages and pitfalls in the application of mixed-model association methods. Nat Genet.

[CR81] Zhu Z, Bakshi A, Vinkhuyzen AA, Hemani G, Lee SH, Nolte IM, van Vliet-Ostaptchouk JV, Snieder H, LifeLines Cohort S (2015). Dominance genetic variation contributes little to the missing heritability for human complex traits. Am J Hum Genet.

