# Supplementary material for: Genome-wide association studies for corneal and refractive astigmatism in UK Biobank demonstrate a shared role for myopia susceptibility loci
Source: Hum Genet. 2018 Oct 10;137(11):881–96. doi: 10.1007/s00439-018-1942-8 (PMC6267700; doi:10.1007/s00439-018-1942-8)
Supplement: Supplementary file 1 — Supplementary material 1 (PDF 1308 KB) [file 439_2018_1942_MOESM1_ESM.pdf]

SUPPLEMENTARY MATERIALS

Genome-wide association studies for corneal and refractive astigmatism in UK Biobank demonstrate a shared role for myopia susceptibility loci

Rupal L. Shah, Jeremy A. Guggenheim, UK Biobank Eye and Vision Consortium

Online Resource	Title	Page
1	Corneal astigmatism and refractive astigmatism as a function of age in the analysed UK Biobank sample.	S2
2	Estimates of SNP-heritability (h^2_{SNP}) for corneal astigmatism, refractive astigmatism and spherical equivalent.	S3
3	Partitioning trait variance into additive, dominance and environmental components using GREMLd.	S4
4	Summary of GWAS undertaken.	S4
5	Conditional analyses performed using summary statistics and conditioning on lead marker – for genome-wide significant associated loci from Corneal Astigmatism GWAS.	S5
6	Conditional analyses performed using summary statistics and conditioning on lead marker – for genome-wide significant associated loci from Refractive Astigmatism GWAS.	S5
7	The 10 markers demonstrating strongest association in GWAS for Corneal Astigmatism analysed as a continuous trait with PLINK 2.0.	S6
8	The 10 markers demonstrating strongest association in GWAS for Corneal Astigmatism analysed as a dichotomous trait (cases ≥1.00D) with PLINK 2.0.	S6
9	The 10 markers demonstrating strongest association in GWAS for Refractive Astigmatism analysed as a continuous trait with PLINK 2.0.	S7
10	The 10 markers demonstrating strongest association in GWAS for Refractive Astigmatism analysed as a dichotomous trait (cases ≥1.00D) with PLINK 2.0.	S7
11	Manhattan plots for GWAS of Corneal and Refractive Astigmatism using PLINK 2.0 as continuous and dichotomous (cases ≥1.00D) traits.	S8
12	Quantile-Quantile plots from GWAS of Corneal and Refractive Astigmatism using PLINK 2.0 as continuous and dichotomous (cases ≥1.00D) traits.	S9
13	Tests for a difference in genetic effect size in male vs. female participants for the genome-wide significant loci for corneal astigmatism analysed as a continuous trait in unrelated participants.	S10
14	Tests for a difference in genetic effect size in male vs. female participants for the genome-wide significant loci for refractive astigmatism analysed as a continuous trait in unrelated participants.	S10
15	The 10 genes demonstrating strongest association from the MAGMA gene-based association test for Corneal Astigmatism as a continuous trait.	S11
16	The 10 genes demonstrating strongest association from the MAGMA gene-based association test for Refractive Astigmatism as a continuous trait.	S12

Online Resource 1. Corneal astigmatism and refractive astigmatism as a function of age in the analysed UK Biobank sample. The width of "violins" corresponds to the distribution of the trait, weighted by the sample size of the age category relative to the full sample. The solid black circle indicates the median and the white rectangle indicates the 25th to 75th percentile of the data.

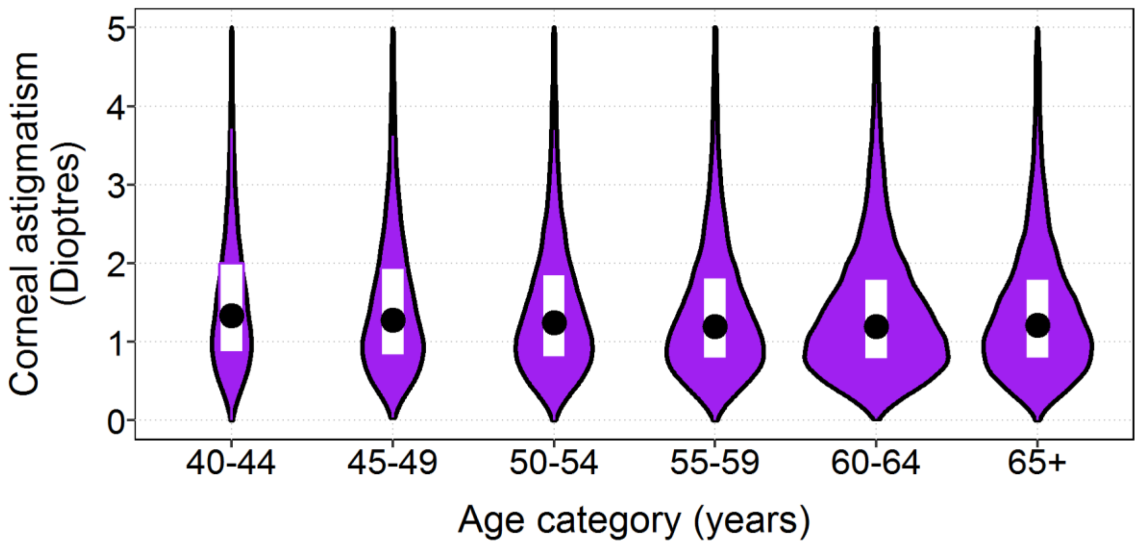

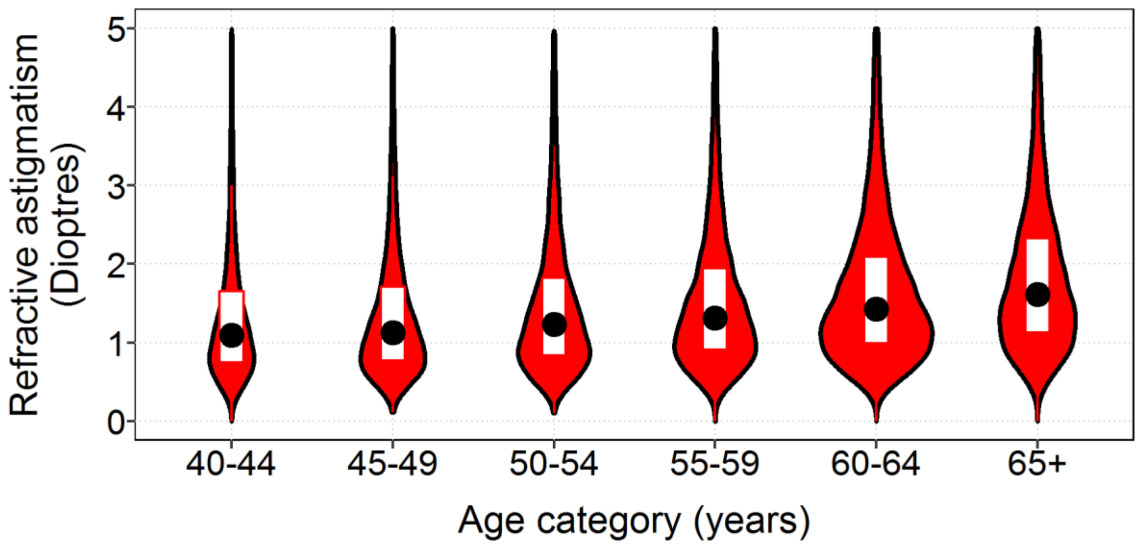

Online Resource 2. Estimates of SNP-heritability (h^2_{SNP}) for corneal astigmatism, refractive astigmatism and spherical equivalent. Analyses were conducted using GCTA-GREML. h^2_{SNP} = SNP-heritability; SE = standard error; P-value = test of the null hypothesis (h^2_{SNP} = 0).

Trait	Case Threshold (D)	Case Prevalence		h^2_{SNP}	SE	P-value
		Population	Sample			
**Corneal Astigmatism (N = 27,707)**	Continuous	-	-	0.061	0.021	1.19×10^{-3}
	0.50	0.72	0.70	0.094	0.036	3.53×10^{-3}
	0.75	0.46	0.45	0.053	0.033	0.051
	1.00	0.28	0.27	0.086	0.038	9.86×10^{-3}
	1.25	0.17	0.17	0.042	0.046	0.175
	1.50	0.11	0.10	0.000	0.057	0.500
**Refractive Astigmatism (N = 28,378)**	Continuous	-	-	0.046	0.020	7.74×10^{-3}
	0.50	0.73	0.71	0.015	0.035	0.332
	0.75	0.47	0.45	0.091	0.032	2.00×10^{-3}
	1.00	0.30	0.28	0.105	0.036	1.45×10^{-3}
	1.25	0.19	0.18	0.158	0.045	1.24×10^{-4}
	1.50	0.12	0.11	0.057	0.055	0.143
**Spherical Equivalent (N = 28,378)**	Continuous	-	-	0.387	0.022	5.05×10^{-85}
	-0.50	0.35	0.33	0.462	0.036	2.61×10^{-43}
	-0.75	0.30	0.29	0.469	0.038	1.74×10^{-41}
	-1.00	0.27	0.27	0.491	0.039	7.04×10^{-42}
	-1.25	0.25	0.24	0.463	0.040	1.30×10^{-34}
	-1.50	0.23	0.22	0.476	0.042	2.20×10^{-33}

Online Resource 3. Partitioning trait variance into additive, dominance and environmental components using GREMLd

Trait	Sample Size	Additive	95% CI	Dominance	95% CI	Environment
**Corneal Astigmatism**	27,714	0.056	0.015-0.096	0.000	0.000-0.036	0.944
**Refractive Astigmatism**	28,378	0.046	0.007-0.085	0.000	0.000-0.035	0.954
**Spherical Equivalent**	28,378	0.368	0.326-0.411	0.000	0.000-0.033	0.632

Online Resource 4. Summary of GWAS undertaken. λ_{GC} = genomic inflation factor; λ_{LDSC} = intercept from LD Score regression. The number of independent loci was identified using GCTA-COJO.

Trait		Method	No. of Markers	Sample Size	λ_{GC}	λ_{LDSC}	No. of Genome-wide Significant Markers ($P < 5 \times 10^{-8}$)	No. of Independent Genome-wide Significant Loci
**Corneal Astigmatism**	Continuous	BOLT-LMM	5,901,671	86,335	1.094	1.023	89	4
	Continuous	PLINK 2.0	5,921,785	69,140	1.057	1.020	54	4
	Cases ≥1.00D	PLINK 2.0	5,921,785	69,140	1.030	1.001	49	3
**Refractive Astigmatism**	Continuous	BOLT-LMM	5,900,115	88,005	1.045	1.005	45	4
	Continuous	PLINK 2.0	5,919,636	70,505	1.042	1.003	13	2
	Cases ≥1.00D	PLINK 2.0	5,919,636	70,505	1.016	0.997	1	1

Online Resource 5. Conditional analyses performed using summary statistics and conditioning on lead marker – for genome-wide significant associated loci from Corneal Astigmatism GWAS. GCTA-COJO Lead Variant: lead variant within ±1 Mb of *GWAS Lead Marker* location

Chromosome	GWAS Lead Marker	P-value	GCTA-COJO Lead Marker	P-value
1	rs12032649	5.00×10^{-14}	NA (co-linearity error)	NA
6	rs196052	7.80×10^{-11}	rs114074527	1.84×10^{-4}
15	rs1129038	1.10×10^{-15}	rs1800407	9.88×10^{-6}
17	rs62075722	2.20×10^{-13}	rs113458760	3.23×10^{-4}

Online Resource 6. Conditional analyses performed using summary statistics and conditioning on lead marker – for genome-wide significant associated loci from Refractive Astigmatism GWAS. GCTA-COJO Lead Variant: lead variant within ±1 Mb of *GWAS Lead Marker* location.

Chromosome	GWAS Lead Marker	P-value	GCTA-COJO Lead Marker	P-value
6	rs12196123	1.60×10^{-15}	rs148281502	2.29×10^{-4}
15	rs1129038	2.30×10^{-11}	rs1800407	9.03×10^{-15}
17	rs34635363	2.00×10^{-9}	rs75248478	9.17×10^{-4}

Online Resource 7. The 10 markers demonstrating strongest association in GWAS for Corneal Astigmatism analysed as a continuous trait with PLINK 2.0. NB: markers within ±500 kb of listed (lead) marker are not included in this list.

Marker	Chromosome	Position	Effect Allele	Reference Allele	Effect (SE)	P-value	Nearest Gene
rs1129038	15	28356859	T	C	0.026 (0.004)	5.73×10^{-12}	*HERC2*
rs12032649	1	219778959	G	T	0.021 (0.003)	6.60×10^{-11}	*ZC3H11B*
rs34635363	17	79549250	A	G	-0.021 (0.003)	7.09×10^{-11}	*NPLOC4*
rs196052	6	22057200	A	T	-0.018 (0.003)	3.53×10^{-8}	*LINC00340*
rs138016380	10	34449466	A	C	0.050 (0.010)	1.40×10^{-6}	*PARD3*
rs142566675	3	35248521	A	C	0.077 (0.016)	1.51×10^{-6}	*LOC101928135*
rs75380963	3	181554299	T	C	0.079 (0.017)	2.29×10^{-6}	*LOC101929598*
rs115822495	6	15681708	T	C	-0.072 (0.015)	2.94×10^{-6}	*DTNBP1*
rs11955682	5	11416298	T	G	0.047 (0.010)	3.15×10^{-6}	*CTNND2*
rs11639295	15	67460757	T	C	-0.016 (0.003)	3.24×10^{-6}	*SMAD3*

Online Resource 8. The 10 markers demonstrating strongest association in GWAS for Corneal Astigmatism analysed as a dichotomous trait (cases ≥1.00D) with PLINK 2.0. NB: markers within ±500 kb of listed (lead) marker are not included in this list.

Marker	Chromosome	Position	Effect Allele	Reference Allele	Odds Ratio (95% CI)	P-value	Nearest Gene
rs72629670	1	219768131	G	A	0.925 (0.901-0.950)	3.95×10^{-10}	*ZC3H11B*
rs62075722	17	79611271	G	A	1.075 (1.051-1.100)	8.14×10^{-9}	*TSPAN10*
rs12913832	15	28365618	G	A	0.920 (0.891-0.950)	2.96×10^{-8}	*HERC2*
rs11238956	10	44749854	C	T	0.937 (0.912-0.962)	2.89×10^{-7}	*C10orf142*
rs1623169	11	128584320	T	C	1.088 (1.056-1.121)	3.80×10^{-7}	*FLI1*
rs6434068	2	153357541	C	G	1.063 (1.039-1.088)	6.18×10^{-7}	*FMNL2*
rs11639295	15	67460757	T	C	1.069 (1.042-1.095)	8.57×10^{-7}	*SMAD3*
rs2763272	6	168832091	T	C	0.932 (0.904-0.960)	1.19×10^{-6}	*SMOC2*
rs115822495	6	15681708	T	C	1.343 (1.219-1.468)	3.57×10^{-6}	*DTNBP1*
rs10223780	6	62865025	G	C	1.059 (1.035-1.083)	3.90×10^{-6}	*KHDRBS2*

Online Resource 9. The 10 markers demonstrating strongest association in GWAS for Refractive Astigmatism analysed as a continuous trait with PLINK 2.0. NB: markers within ±500 kb of listed (lead) marker are not included in this list.

Marker	Chromosome	Position	Effect Allele	Reference Allele	Effect (SE)	P-value	Nearest Gene
rs12196123	6	22082263	T	C	-0.022 (0.003)	1.07×10^{-11}	*LINC00340*
rs1129038	15	28356859	T	C	-0.024 (0.004)	3.63×10^{-10}	*HERC2*
rs10177414	2	228211470	C	T	-0.018 (0.003)	5.95×10^{-8}	*MFF*
rs34635363	17	79549250	A	G	-0.018 (0.003)	1.75×10^{-7}	*NPLOC4*
rs11244084	9	136191010	T	C	0.032 (0.006)	1.78×10^{-7}	*LCN1P2*
rs139743	22	25299429	G	A	-0.017 (0.003)	2.03×10^{-7}	*SGSM1*
rs116771750	1	219699050	C	T	-0.046 (0.009)	2.11×10^{-7}	*ZC3H11B*
rs2072970	20	40112488	A	G	-0.017 (0.003)	7.93×10^{-7}	*CHD6*
rs55939894	3	7963288	G	A	-0.020 (0.004)	9.09×10^{-7}	*LOC101927394*
rs17172445	7	55189215	T	G	-0.049 (0.010)	9.43×10^{-7}	*EGFR*

Online Resource 10. The 10 markers demonstrating strongest association in GWAS for Refractive Astigmatism analysed as a dichotomous trait (cases ≥1.00D) with PLINK 2.0. NB: markers within ±500 kb of listed (lead) marker are not included in this list.

Marker	Chromosome	Position	Effect Allele	Reference Allele	Odds Ratio (95% CI)	P-value	Nearest Gene
rs10946507	6	22100367	A	G	0.930 (0.906-0.954)	2.15×10^{-9}	*LINC00340*
rs10177414	2	228211470	C	T	0.942 (0.918-0.966)	1.34×10^{-6}	*MFF*
rs11244084	9	136191010	T	C	0.898 (0.853-0.942)	2.07×10^{-6}	*LCN1P2*
rs73430835	7	111628421	C	T	0.777 (0.673-0.882)	2.19×10^{-6}	*DOCK4*
rs2026781	10	133841039	C	G	1.067 (1.040-1.094)	2.62×10^{-6}	*JAKMIP3*
rs114330092	5	178661923	T	G	0.866 (0.807-0.926)	2.81×10^{-6}	*ADAMTS2*
rs12051285	16	7460115	G	T	1.057 (1.034-1.081)	4.61×10^{-6}	*RBFOX1*
rs192526513	7	44989410	A	G	1.245 (1.150-1.340)	5.54×10^{-6}	*MYO1G*
rs151198248	20	42389025	T	C	1.335 (1.210-1.460)	6.19×10^{-6}	*LOC101927200*
rs76638496	8	82917849	A	G	0.829 (0.748-0.911)	6.39×10^{-6}	*SNX16*

Online Resource 11. Manhattan plots for GWAS of Corneal and Refractive Astigmatism using PLINK 2.0 as continuous and dichotomous (cases ≥1.00D) traits. A: Corneal Astigmatism (continuous trait); **B**: Corneal Astigmatism (dichotomous trait); **C**: Refractive Astigmatism (continuous trait); **D**: Refractive Astigmatism (dichotomous trait). All panels: Red line indicates $P = 5 \times 10^{-8}$; blue line indicates $P = 1 \times 10^{-5}$.

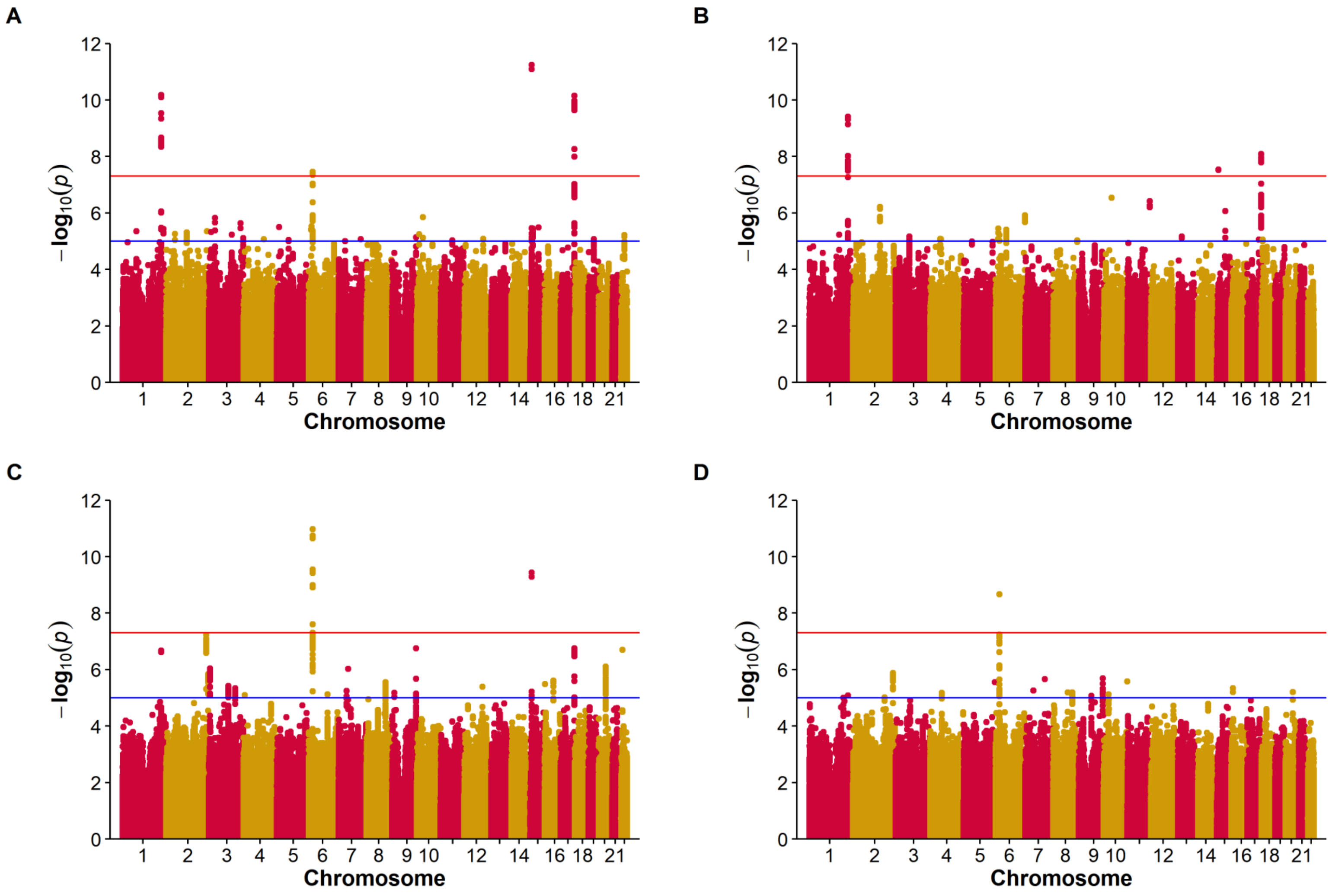

Online Resource 12. Quantile-Quantile plots from GWAS of Corneal and Refractive Astigmatism using PLINK 2.0 as continuous and dichotomous (cases ≥1.00D) traits. A: Corneal Astigmatism (continuous trait); **B**: Corneal Astigmatism (dichotomous trait); **C**: Refractive Astigmatism (continuous trait); **D**: Refractive Astigmatism (dichotomous trait). All panels: Y-axis shows observed negative $\log_{10}$ p-values and X-axis shows expected negative $\log_{10}$ p-values according to the null hypothesis of no genetic association. Red line: line of unity (observed = expected).

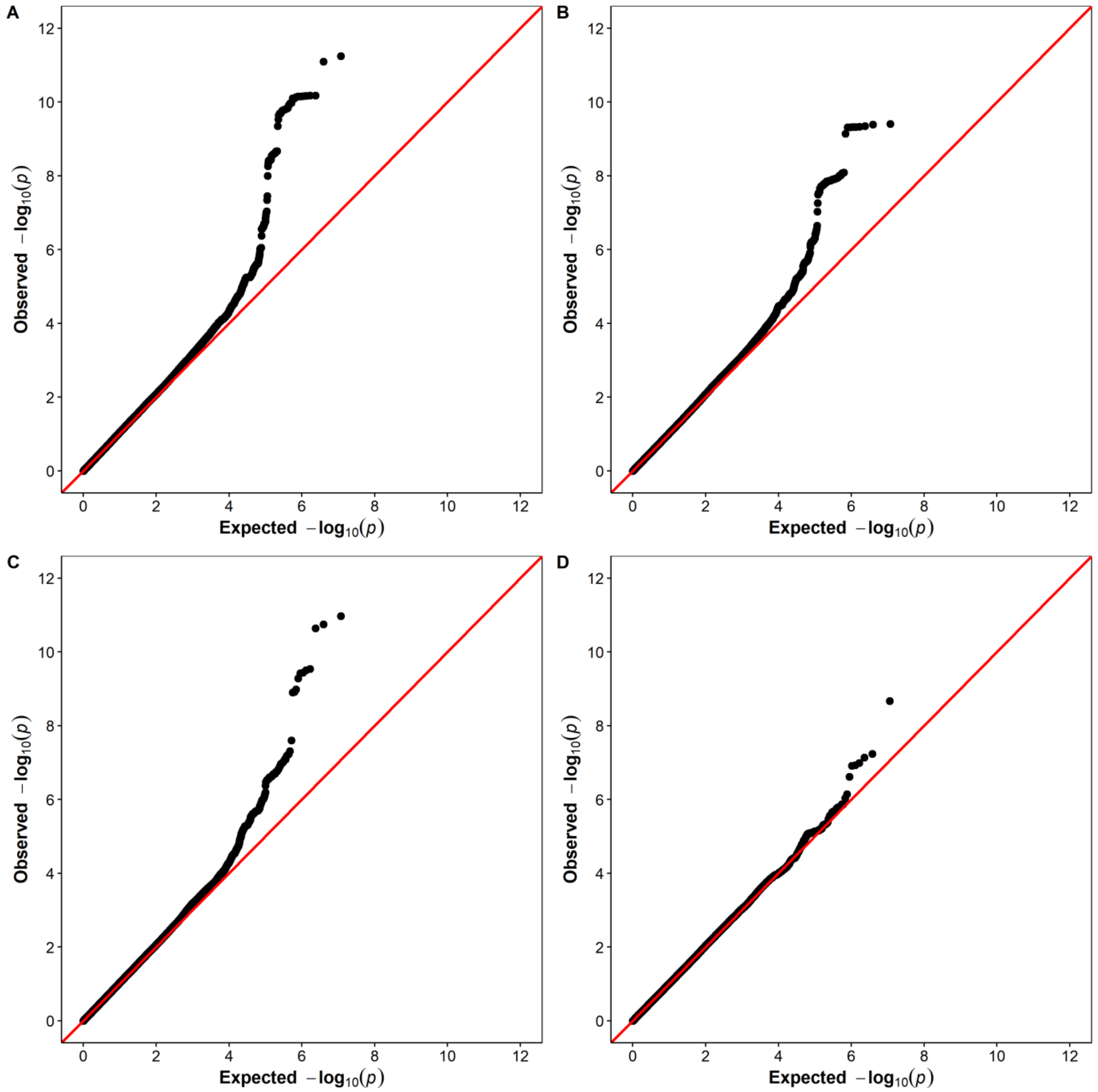

Online Resource 13. Tests for a difference in genetic effect size in male vs. female participants for the genome-wide significant loci for corneal astigmatism analysed as a continuous trait in unrelated participants. Age, spherical equivalent refractive error, and a binary indicator for genotyping array were included as covariates.

Marker	Effect Allele	Female participants (n=37,377)		Male participants (n=31,763)		Difference in effect size in females vs. males P-value	Nearest Gene
		Beta (SE) in Dioptres	P-value	Beta (SE) in Dioptres	P-value		
rs1129038	C	-0.034 (0.005)	3.59E-11	-0.017 (0.006)	3.03E-03	0.024	*HERC2*
rs12032649	T	-0.026 (0.004)	5.84E-09	-0.017 (0.005)	6.22E-04	0.167	*ZC3H11B*
rs62075722	A	0.023 (0.004)	2.03E-07	0.018 (0.005)	1.59E-04	0.479	*TSPAN10*
rs196052	T	0.019 (0.004)	1.06E-05	0.017 (0.005)	5.42E-04	0.682	*LINC00340*

Online Resource 14. Tests for a difference in genetic effect size in male vs. female participants for the genome-wide significant loci for refractive astigmatism analysed as a continuous trait in unrelated participants. Age, spherical equivalent refractive error, and a binary indicator for genotyping array were included as covariates.

Marker	Effect Allele	Female participants (n=37,379)		Male participants (n=33,126)		Difference in effect size in females vs. males P-value	Nearest Gene
		Beta (SE) in Dioptres	P-value	Beta (SE) in Dioptres	P-value		
rs12196123	C	-0.017 (0.004)	5.26E-05	-0.028 (0.005)	2.37E-08	0.121	*LINC00340*
rs1129038	C	-0.027 (0.005)	1.49E-07	-0.021 (0.006)	3.46E-04	0.456	*HERC2*
rs34635363	A	0.016 (0.004)	4.10E-04	0.020 (0.005)	9.63E-05	0.533	*NPLOC4*

Online Resource 15. The 10 genes demonstrating strongest association from the MAGMA gene-based association test for Corneal Astigmatism as a continuous trait. Start and stop positions listed include ±50 kb flanking regions; nSNPs = number of variants included in gene region; Z-Statistic = gene-based test statistic; P-value = obtained from Z-Statistic under the assumption of a normally distributed model; FDR = false discovery rate.

Gene	Chromosome	Start	Stop	nSNPs	Z Statistic	P-value	FDR	Bonferroni Adjusted P
NPLOC4	17	79473913	79654138	716	6.34	1.15×10^{-10}	2.10×10^{-6}	2.10×10^{-6}
TSPAN10	17	79559349	79665779	485	5.71	5.75×10^{-9}	5.27×10^{-5}	1.05×10^{-4}
PDE6G	17	79567489	79673607	479	5.46	2.36×10^{-8}	1.44×10^{-6}	4.33×10^{-4}
HERC2	15	28306183	28617313	185	5.33	5.01×10^{-8}	2.30×10^{-4}	9.19×10^{-4}
CCDC137	17	79583761	79690937	470	5.24	7.88×10^{-8}	2.89×10^{-4}	1.45×10^{-3}
OXLD1	17	79582066	79683618	476	5.16	1.24×10^{-7}	3.78×10^{-4}	2.27×10^{-3}
PDGFRA	4	55045264	55214412	324	5.07	1.99×10^{-7}	5.21×10^{-4}	3.65×10^{-3}
C17orf70	17	79456911	79569429	402	4.93	4.08×10^{-7}	9.34×10^{-4}	7.47×10^{-3}
HGS	17	79600962	79719151	423	4.82	7.24×10^{-7}	1.48×10^{-3}	0.013
ARL16	17	79598224	79700954	437	4.68	1.47×10^{-6}	2.69×10^{-3}	0.027

Online Resource 16. The 10 genes demonstrating strongest association from the MAGMA gene-based association test for Refractive Astigmatism as a continuous trait. Start and stop positions listed include ±50 kb flanking regions; nSNPs = number of variants included in gene region; Z-Statistic = gene-based test statistic; P-value = obtained from Z-Statistic under the assumption of a normally distributed model; FDR = false discovery rate.

Gene	Chromosome	Start	Stop	nSNPs	Z Statistic	P-value	FDR	Bonferroni Adjusted P
TMEM211	22	25281208	25385314	144	4.90	4.72×10^{-7}	4.96×10^{-3}	8.65×10^{-3}
PROX1	1	214111278	214264853	278	4.88	5.41×10^{-7}	4.96×10^{-3}	9.92×10^{-3}
HERC2	15	28306183	28617313	186	4.76	9.86×10^{-7}	5.00×10^{-3}	0.018
TSPAN10	17	79559349	79665779	484	4.74	1.09×10^{-6}	5.00×10^{-3}	0.020
NPLOC4	17	79473913	79654138	717	4.60	2.15×10^{-6}	5.69×10^{-3}	0.039
PDE6G	17	79567489	79673607	478	4.60	2.15×10^{-6}	5.69×10^{-3}	0.039
PLAUR	19	44100247	44224498	254	4.59	2.17×10^{-6}	5.69×10^{-3}	0.040
OXLD1	17	79582066	79683618	475	4.38	5.87×10^{-6}	0.013	0.108
CCDC137	17	79583761	79690937	469	4.35	6.71×10^{-6}	0.014	0.123
VAX2	2	71077720	71210576	281	4.33	7.59×10^{-6}	0.014	0.139
